# Hybrid Targeted/Untargeted Screening Method for the Determination of Wildfire and Water-Soluble Organic Tracers in Ice Cores and Snow

**DOI:** 10.1021/acs.analchem.3c01852

**Published:** 2023-07-18

**Authors:** François Burgay, Daniil Salionov, Carla Jennifer Huber, Thomas Singer, Anja Eichler, Florian Ungeheuer, Alexander Vogel, Margit Schwikowski, Saša Bjelić

**Affiliations:** † Laboratory of Environmental Chemistry (LUC), Paul Scherrer Institut, 5232 Villigen PSI, Switzerland; ‡ Oeschger Centre for Climate Change Research, University of Bern, 3012 Bern, Switzerland; § Bioenergy and Catalysis Laboratory (LBK), Paul Scherrer Institut, 5232 Villigen PSI, Switzerland; ∥ Department of Chemistry, Biochemistry and Pharmaceutical Sciences, University of Bern, 3012 Bern, Switzerland; ⊥ Institute for Atmospheric and Environmental Sciences (IAU), Goethe Universität, 60438 Frankfurt am Main, Germany

## Abstract

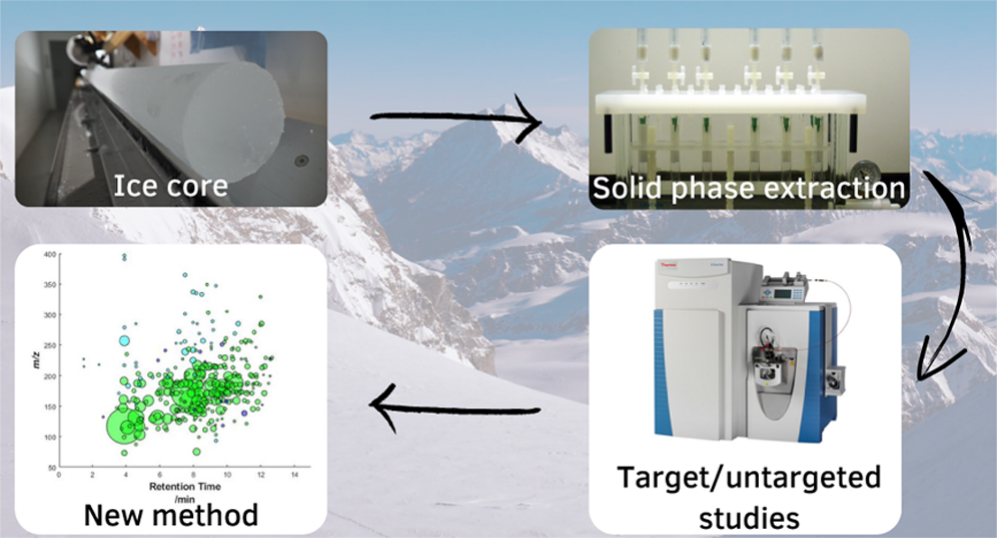

Wildfires can influence the earth’s radiative forcing through the emission of biomass-burning aerosols. To better constrain the impacts of wildfires on climate and understand their evolution under future climate scenarios, reconstructing their chemical nature, assessing their past variability, and evaluating their influence on the atmospheric composition are essential. Ice cores are unique to perform such reconstructions representing archives not only of past biomass-burning events but also of concurrent climate and environmental changes. Here, we present a novel methodology for the quantification of five biomass-burning proxies (syringic acid, vanillic acid, vanillin, syringaldehyde, and *p*-hydroxybenzoic acid) and one biogenic emission proxy (pinic acid) using solid phase extraction (SPE) and ultrahigh-performance liquid chromatography coupled with high-resolution mass spectrometry. This method was also optimized for untargeted screening analysis to gain a broader knowledge about the chemical composition of organic aerosols in ice and snow samples. The method provides low detection limits (0.003–0.012 ng g^–1^), high recoveries (74 ± 10%), and excellent reproducibility, allowing the quantification of the six proxies and the identification of 313 different molecules, mainly constituted by carbon, hydrogen, and oxygen. The effectiveness of two different sample storage strategies, i.e., re-freezing of previously molten ice samples and freezing of previously loaded SPE cartridges, was also assessed, showing that the latter approach provides more reproducible results.

## 1 Introduction

Forest fires are a key component of the earth system and can influence earth’s radiative forcing^1,2^ by emitting large amounts of aerosol particles and gases into the atmosphere, mainly black carbon (>86% of global emissions) and particulate organic carbon (up to 39% of global emissions).^3,4^ Predicting the global wildfire evolution under future climate scenarios is complicated due to fire’s heterogeneous geographical distribution and evolution.^5^ To reduce these uncertainties, it is essential to unravel the wildfire interactions with vegetation types, human activities, and climate. Ice cores represent a well-suited environmental archive to perform such studies over long timescales since the biomass-burning fingerprint is preserved in their stratigraphy and can be related to other climate and environmental variables (e.g., temperature, precipitation, and vegetation types).^3,6,7^ Commonly used biomass-burning proxies in ice cores are inorganic ions such as NH_4_^+^ and K^+^^8^ or black carbon.^9^ However, these tracers are not unambiguous since they can originate from other emission sources (e.g., mineral dust and biogenic and anthropogenic emissions), complicating the identification of biomass-burning signals.^6,10^ To overcome these difficulties, specific organic fire proxies have been proposed,^11^ such as levoglucosan, a cellulose degradation product. Despite being widely studied at different ice-core locations,^12−14^ levoglucosan is aspecific toward the kind of vegetation that burns.^15^ To overcome this limitation, new studies focused on lignin degradation products. Lignin is a biopolymer that constitutes 20–30% of the dry wood mass.^16^ Depending on the vegetation type, lignin can be enriched in one of its constituents. That is why, during conifer (gymnosperms) combustion, vanillic-like compounds are produced, while during flowering plant (angiosperms) combustion, syringic-like species are emitted, and during grass (graminae) combustion, *p*-hydroxybenzoic acid is produced.^17^ Once emitted into the atmosphere, these compounds can experience photochemical degradation and/or heterogeneous oxidation pathways through the reaction with O_3_ and OH radicals.^18^ Methoxyphenols containing an aldehyde functional group can be oxidized to carboxylic compounds. Therefore, the evaluation of the ratios between aldehydes and their corresponding acids is a useful tool to understand the degree of aerosol transformation and aging during transport.^19^ To date, only a few of these compounds have been investigated in ice-core samples.^20−25^

Identifying individual wildfire proxies alone is still insufficient to understand the links between wildfires and climate and to investigate how forest fires can influence atmospheric chemistry. Recent advances in high-resolution mass spectrometry and the availability of untargeted screening (NTS) workflows have unlocked the possibility to largely increase the spectrum of detectable molecules, up to thousands of different compounds from single environmental samples.^26^ Through the exploration of a wider chemical space, NTS applied to ice cores enables a better understanding of the impact of wildfires on the chemical composition and on the oxidizing capacity of the atmosphere on a regional scale.^27^

In this study, we developed a novel methodology that couples a targeted approach for quantifying biomass-burning proxies (i.e., syringic acid, vanillic acid, vanillin, syringaldehyde, and *p*-hydroxybenzoic acid), which are crucial for identifying wildfire horizons in ice cores, with a novel NTS approach for the identification of secondary organic aerosol tracers. The methodology is also designed to quantify pinic acid, a biogenic emission proxy, and will contribute to gaining knowledge on the wildfires’ influence on the atmospheric chemical properties.

Last, considering that a fundamental step between sample collection and analysis is ensuring analyte preservation and preventing or minimizing any physical or chemical change (e.g., adsorption, diffusion, volatilization, oxidation, and microbial degradation), we assessed organic tracer preservation after melting of the ice/snow samples. We investigated two main sample storage strategies that involve the re-freezing of previously molten ice samples^28−30^ and the physical adsorption on a solid phase.^31^ Previous studies performed on different chemicals (drugs) and matrices (wastewater) showed divergent results,^32,33^ indicating that case-specific investigations are needed to assess analyte’s preservation during sample storage.

## 2 Experimental Section

### 2.1 Chemicals, Reagents, and Sample Preparation

Details on the standards used for the method development, cartridges, and solvents are reported in the Supporting Information (SI1). For method development, *n* = 2 bulk snow samples from the high-altitude research station Jungfraujoch (3460 m.a.s.l) and *n* = 15 ice-core samples from Colle Gnifetti (4450 m.a.s.l.) were used. For the application of the method, *n* = 10 ice-core samples from Grand Combin (4123 m.a.s.l.) and *n* = 1 ice-core sample from the Belukha glacier (4062 m.a.s.l.) were used. More details about the sampling locations and how the snow and ice-core samples were processed are described in SI2.

### 2.2 Labware Decontamination Procedures

The cut ice-core sections from Belukha and Colle Gnifetti (i.e., method application and development cores, respectively) were stored in 2 L polyethylene (PE) jars that were cleaned as follows: they were filled with ultrapure water (UPW) for at least 24 h, then rinsed with UPW, and refilled with UPW for additional 24 h. This procedure was repeated five times for each PE jar. The glass vials (50 mL, Infochroma, AG) used to store the molten core samples for method development were cleaned according to previously published protocols:^34^ they were baked at 450 °C for 8 h, rinsed with UPW 3× and with methanol 3×, and dried overnight under a Class-1000 laminar flow hood. Once cut, the Grand Combin ice-core sections (i.e., method application core) were stored in 240 mL glass jars (Infochroma AG) that were previously cleaned using the same procedure described above for the 50 mL glass vials. The Jungfraujoch bulk snow samples were collected in a 2 L glass jar that was previously rinsed 5× with UPW, 3× with methanol, and then dried under a Class-1000 laminar flowhood.

The 1.5 mL MS-vials (BG Analytics) used for analyses and the 1.5 mL tubes (Eppendorf) used for the standard solution preparation were 3× rinsed with UPW, ultrasonicated for 20 min at 25 °C, 3× rinsed with UPW, 2× rinsed with methanol, and dried overnight under a Class-1000 laminar flowhood.

### 2.3 Solid Phase Extraction (SPE) Procedure

The pre-concentration conditions were optimized after testing different elution solutions and through the implementation of a decontamination and a counterion step. More details are provided in SI3.

Approximately 300 g of the Colle Gnifetti and Belukha ice-core sections was rinsed with UPW and then molten in a glass vessel under a helium atmosphere.^35^ Once the ice was completely molten, 50 mL aliquots were filtered using a quartz fiber filter (PALLFLEX, Tissuquartz filters 2500QAT-UP, diameter of 47 mm) and collected into the pre-cleaned 50 mL glass vials. Filters were previously baked at 800 °C for 5 h. For the Grand Combin ice core, ≈70 g of the ice sections was molten at room temperature inside the pre-cleaned 240 mL glass jars in a ≈20 °C water bath under a Class-1000 laminar flowhood.

Before SPE, the molten samples were spiked with 75 μL of 40 ng g^–1^ internal standard (*p*-hydroxybenzoic acid-(phenyl-^13^C_6_)) and alkalized with 8 μL of NH_4_OH (25% in UPW) to pH ≈ 10. After 60 min, the samples were pre-concentrated following the SPE procedure described below. To account for possible sources of contamination, 14 procedural blanks were prepared from 30 g of frozen UPW and treated as samples.

The SPE cartridges (Strong Anionic Exchange, MAX, 1 mL, 10 mg bed weight, Waters) were conditioned with 1 mL of methanol followed by 5 mL of UPW. To minimize any possible contamination from the cartridges, we introduced a decontamination step consisting of 500 μL of a 0.16 M HCl solution in methanol followed by 2 mL of UPW. To enhance the selectivity of the cartridges toward the analytes, the counterion was changed from chloride to formate using 500 μL of a 2% formic acid solution in UPW followed by 2 mL of UPW. Molten ice samples (50 mL) were loaded onto the cartridges using PTFE transfer tubes at a flow rate of 1–2 mL min^–1^. To avoid external contamination, the cartridges’ tops, as well as the glass vials, were covered with an aluminum foil during the loading step. Then, the cartridges were dried under vacuum for 5 min. To avoid any cross-contamination, the PTFE transfer tubes were changed after each sample.

In the test experiments involving the cartridge freezing (Section 2.6, SI5), the cartridges were wrapped in two aluminum foils after drying and stored at −20 °C. Before elution, these cartridges were thawed at room temperature under a Class-1000 laminar flowhood for ≈30 min. For all other approaches, cartridges were directly eluted after the loading step.

The elution of the analytes from the cartridges was carried out using 3 × 250 μL of a 5% formic acid solution in methanol at a flow rate of 1 mL min^–1^ in pre-cleaned 1.5 mL vials. To avoid cross-contamination, the SPE manifold liners were disposed and changed after each sample. The obtained 750 μL eluates were pre-concentrated to ≈40 μL at 30 °C under a gentle N_2_ flow (Reacti-Vap Evaporator, Thermo Fischer Scientific) and then retaken with 475 μL of UPW before analysis. Subsequently, 25 μL of 1.4 μg g^–1^ vanillin-(phenyl-^13^C_6_) solution (10% v/v MeOH/UPW) was added as an additional internal standard to monitor the instrument performances.

### 2.4 Instrumental Analysis

For analyses, samples were transferred to a thermostated autosampler (*T* = 10 °C) and analyzed within 24 h with ultrahigh-performance liquid chromatography (Ultimate 3000, Thermo Scientific) equipped with an Acclaim Organic Acid Column (3 μm, 2.1 × 150 mm, Thermo Scientific, operated at 50 °C) coupled with high-resolution mass spectrometry (UHPLC-HRMS, Q Exactive Focus, Thermo Scientific). The instrumental parameters were optimized after testing different chromatographic columns, different elution gradients, and different concentrations of the eluent modifier (i.e., formic acid). More details are provided in SI4. The final, optimized setup is given in the following paragraphs.

The injection volume was 20 μL. Chromatographic separation was obtained using a mobile phase consisting of 0.01% formic acid, 1% acetonitrile, and 1% methanol in water (v/v/v, eluent A) and methanol (eluent B) with a flow rate of 0.2 mL min^–1^. The binary elution program was as follows: 0–12 min linearly increasing gradient from 8% to 90% of B, 12–15 min isocratic elution at 90% B. The ionization of compounds was performed using a heated electrospray ionization source operating in negative ionization mode. Data acquisition was performed in Full MS with a scan range from 70 to 1000 mass-to-charge ratio (*m/z*). The chromatograms for the six targeted species at a concentration of 10 ng g^–1^ are shown in Figure S2.

The instrumental conditions for electrospray ionization were as follows: sheath gas (N_2_) 35 a.u., auxiliary gas (N_2_) 10 a.u., probe heater temperature 300 °C, capillary temperature 280 °C, and capillary voltage 2.5 kV. The MS-data were recorded in centroid mode with lock mass at *m/z* 112.98563 (sodium formate cluster). Resolution at *m/z* = 200 was 7 × 10^4^. Data analysis for the identification and quantification of the targeted species was performed using the XCalibur software v. 4.1 (Thermo Scientific), while Compound Discoverer v. 3.3 (Thermo Scientific) was used for the NTS study (SI6).

### 2.5 Evaluation of the Method Performances

#### 2.5.1 Targeted Approach

To evaluate the performances of the developed methodology for the targeted approach, we determined instrumental accuracy and precision, instrumental limits of detection, methodological limits of detection, matrix effect, recovery, and reproducibility.

Instrumental accuracy is expressed as (*O* – *T*)/*T* %, where *O* is the determined value and *T* is the concentration of the quality control (QC) samples. Instrumental precision (*n* = 3) is expressed as the relative standard deviation (%RSD) of the QC samples. To determine these parameters, we prepared QC samples in UPW at 1 ng g^–1^ (*n* = 3) and 10 ng g^–1^ (*n* = 3).

We investigated both instrumental (i.e., the lowest amount of analyte detectable by the instrument) and methodological (i.e., including the possible sample contamination during the analytical procedure) limits of detection. The instrumental limits of detection were calculated as 3.3 × σ/S where σ is the standard deviation of the regression line (*n* = 3) and *S* is the slope, while the methodological limits of detection (MDL) are quantified as three times the standard deviation of the procedural blanks (*n* = 14).

The matrix effect is defined here as the difference in terms of response factors between the calibration curves prepared in UPW and the calibration curves prepared in a matrix that mimicked the composition of the SPE eluate (hereafter, the elution matrix) for the six targeted species.^36^ Calibration curves were prepared from 0.5 to 15 ng g^–1^ by sequential dilution of a 5000 mg g^–1^ stock solution (containing syringic acid, syringaldehyde, vanillic acid, vanillin, *p*-hydroxybenzoic acid, and pinic acid; prepared in methanol and stored at −20 °C). To each standard, 50 μL of 1.4 μg g^–1^ vanillin-(phenyl-^13^C_6_) was added. To prepare the elution matrix, we filled the MS-vials with 750 μL of the eluent solution (5% formic acid in methanol), pre-concentrated to ≈40 μL at 30 °C under a gentle N_2_ flow, and finally added UPW, the standard at a known concentration, and ^13^C-vanillin as the internal standard. Each standard was analyzed in triplicate, and three independent sets of standards in UPW and in the elution matrix (i.e., three sets of two calibration curves each) were prepared. The matrix effect was tested by comparing the slope between the two different calibration curves, and it is presented as the difference between the response factor of the calibration curve prepared in UPW and the response factor of the calibration curve prepared in the elution matrix, divided by the response factor for the calibration curve prepared in UPW.

The method recovery was evaluated by extraction and analysis of four sets of 50 mL UPW standard solutions prepared at three different concentrations (at 0.03, 0.1, and 1 ng g^–1^).

Reproducibility was evaluated on a Colle Gnifetti ice-core sample, which was divided into two 30 mL aliquots.

#### 2.5.2 Untargeted Screening Approach

To evaluate the performances of the developed methodology for the NTS approach, we determined instrumental mass accuracy (defined as the difference between measured and theoretical molecular weight of assigned annotation in ppm), instrumental precision, number of identifications, and reproducibility. Reproducibility was evaluated within the freezing test experiments, and it is discussed in Section 3.3.2 in detail. In addition, we also compared the method performances with a previous method used for ice core analyses.^28^

### 2.6 Freezing Tests

Using the developed methodology, we evaluated the effectiveness of two different storage approaches that might be used when ice or snow samples cannot be analyzed directly after melting: (a) re-freezing the samples in glass vials and (b) re-freezing the samples after they are loaded onto SPE cartridges. Tests were performed at two different spiked concentrations (i.e., ≈0.03 ng g^–1^ and ≈0.1 ng g^–1^) following both a targeted and an untargeted screening approach. The areas of the compounds in the unfrozen and frozen samples were compared to evaluate analyte loss and to define the best sample storage strategy. The full procedure for these experiments is described in details in SI5.

## 3 Results and Discussion

In this section, we explore the method performances and evaluate them in comparison to other similar methods from previous studies. The application of the method is tested for both the targeted and untargeted screening approaches on Grand Combin and Belukha samples, respectively. Furthermore, the method was applied to test two different sample storage strategies to highlight the most suitable approach when already molten ice or snow samples need to be re-frozen.

### 3.1 Method Performances and Application on Real Samples: Targeted Approach

Method validation for the targeted approach was performed evaluating the linearity of the calibration curves, instrumental accuracy, instrumental precision, instrumental limits of detection, matrix effect, recoveries, methodological limits of detection, and reproducibility.

The calibration curves prepared in UPW showed excellent linearity over the range 0.5–15 ng g^–1^ (Figure S3) that covers the expected environmental concentration of the target compounds (after SPE enrichment).

Overall, instrumental accuracy was between −17% and +15%, while instrumental precision was below 4%RSD (Table 1). The instrumental limits of detection ranged between 2.5 and 12 pg per injection for all targeted species, which were comparable to previous studies (Table S3). The methodological limits of detection (MDL) were 0.005 ng g^–1^ (syringic acid), 0.012 ng g^–1^ (vanillic acid), 0.007 ng g^–1^ (vanillin), 0.003 ng g^–1^ (syringaldehyde), 0.007 ng g^–1^ (*p*-hydroxybenzoic acid), and 0.010 ng g^–1^ (pinic acid). MDL are compatible with the expected environmental concentrations of the targeted compounds (Table S4). Achieving such low LOD and MDL enables the possibility to investigate simultaneously all the six targeted species at trace and ultratrace levels in ice cores for the first time.

**Table 1:** Summary of the Method Performances^a^

				instrumental accuracy (%)		instrumental precision (%RSD)					
compound	RT (min)	LoD (pg)	MDL (ng g^–1^)	1 ng g^–1^	10 ng g^–1^	1 ng g^–1^	10 ng g^–1^	recovery (%)	*R*^2^	RF	matrix effect (%)
syringic acid	9.05	12 ± 3	0.005	–10 ± 7	4 ± 1	2 ± 2	0.7 ± 0.5	62 ± 7	0.99	1.1 ± 0.1	6 ± 6
vanillic acid	8.94	7 ± 1	0.012	5 ± 10	0.6 ± 0.6	0.9 ± 0.7	2 ± 1	69 ± 6	0.99	1.0 ± 0.1	9 ± 4
vanillin	9.22	4 ± 1	0.007	0 ± 3	0 ± 2	0.6 ± 0.6	0.9 ± 0.5	67 ± 10	0.99	1.02 ± 0.01	1 ± 1
syringaldehyde	9.30	10 ± 3	0.003	–3 ± 2	2 ± 3	2 ± 1	0.9 ± 0.3	78 ± 5	0.99	0.85 ± 0.04	3 ± 3
*p*-hydroxybenzoic acid	8.58	2.5 ± 0.9	0.007	0 ± 4	1.4 ± 0.8	2 ± 2	0.8 ± 0.2	81 ± 6	0.99	3.8 ± 0.1	1 ± 3
pinic acid	9.03	3 ± 1	0.010	–3 ± 4	1 ± 1	0.8 ± 0.2	0.3 ± 0.2	87 ± 7	0.99	2.2 ± 0.1	2 ± 8

a Retention time (RT) is expressed in min. The LoD is the instrumental limit of detection (*n* = 3). MDL is the methodological method of detection. Instrumental accuracy and precision were calculated at 1 ng g^–1^ (*n* = 3) and 10 ng g^–1^ (*n* = 3). The average recovery calculated at three different concentration levels (0.03, 0.1, and 1 ng g^–1^) is reported. The *R*^2^ and RF (response factor) parameters refer to a calibration curve using ^13^C vanillin as an internal standard (*n* = 3). The matrix effect (in %) was calculated as the difference between the response factor calculated from calibration curves prepared in UPW (*n* = 3) and the response factor calculated from the calibration curves prepared in the SPE elution matrix (*n* = 3), divided by the response factor calculated for the standards prepared in UPW.

Concerning the effects of the SPE elution solution on the ionization efficiency (defined here as the matrix effect), no statistically significant matrix effect was detected (*p*-value >0.05) in the investigated concentration range (0.5 to 15 ng g^–1^), indicating that calibration curves can be prepared in UPW and that the SPE elution solution does not affect the ionization efficiency.

On average, recoveries were 62 ± 7% for syringic acid, 69 ± 6% for vanillic acid, 67 ± 10% for vanillin, 78 ± 5% for syringaldehyde, 81 ± 6% for *p*-hydroxybenzoic acid, and 87 ± 7% for pinic acid (Figure S4). No statistical differences were observed among recoveries performed at different concentration levels (*p*-value >0.05). Thus, recoveries are independent of the concentrations of the compounds in the sample, indicating the robustness of the method for at least two orders of magnitude changes in concentrations (i.e., from 0.03 to 1 ng g^–1^). All the concentration data presented in this manuscript were corrected, taking into account the average recovery presented above.

Reproducibility evaluated on two aliquots from a Colle Gnifetti ice-core sample ranged between 0.6%RSD and 4.1%RSD for all the targeted species except vanillin (32%RSD). This result is consistent with the recovery experiments that showed overall good reproducibility, generally lower than 12%RSD. The high %RSD observed for vanillin is consistent with the high %RSD that was also found during the recovery experiments. The explanation can be linked either to a non-optimal desorption/adsorption on the SPE cartridge or to a different volatilization behavior of vanillin during the evaporation step.

**Figure 1:**
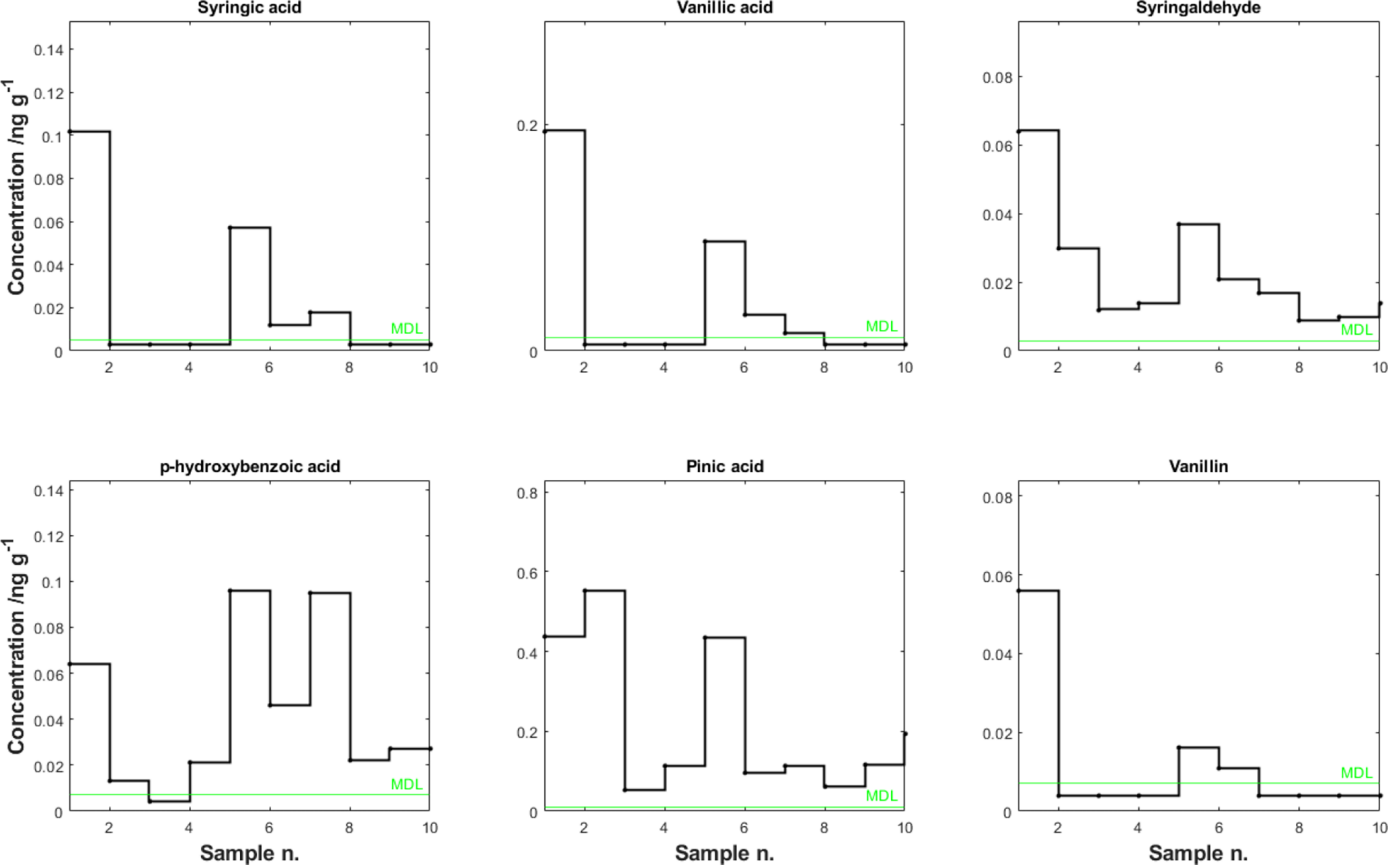
Temporal profile of the six targeted organic species from a section of the Grand Combin ice core. The green line represents the methodological limit of detection (MDL).

The developed method was applied to one Belukha ice-core sample (that was also used for NTS) and to 10 selected samples from the Grand Combin ice core (Figure 1). In the Belukha ice core, we only detected *p*-hydroxybenzoic acid (0.12 ng g^–1^) and pinic acid (1.5 ng g^–1^), while for the Grand Combin samples, we detected all six targeted compounds in at least one sample, indicating the relevance of their quantification in ice samples from a paleoenvironmental perspective. The most abundant species was pinic acid (up to 0.553 ng g^–1^), while the lowest concentrations were found for syringaldehyde (up to 0.064 ng g^–1^) and vanillin (up to 0.056 ng g^–1^). The low observed concentrations for latter compounds emphasize the need for a pre-concentration step to improve the instrumental detection. The environmental interpretation of the Grand Combin ice-core record is beyond the scope of this manuscript and will therefore not be discussed further. The interpretation of the full record, together with the results of the associated NTS analysis, will be discussed on a distinct paper.

### 3.2 Method Performances and Application on Real Samples: Untargeted Screening Approach

Method performances for the NTS were evaluated assessing instrumental mass accuracy, precision, number of identifications, comparison with a previous method for ice core analyses,^28^ and reproducibility.

Instrumental mass accuracies (*n* = 3) were between 2.87 and −2.31 ppm, indicating good instrumental performances that constrained the number of possible candidates for every identified mass. The instrumental precision (*n* = 3) was between −20 and 20%RSD for 90% of the identified compounds.

**Figure 2:**
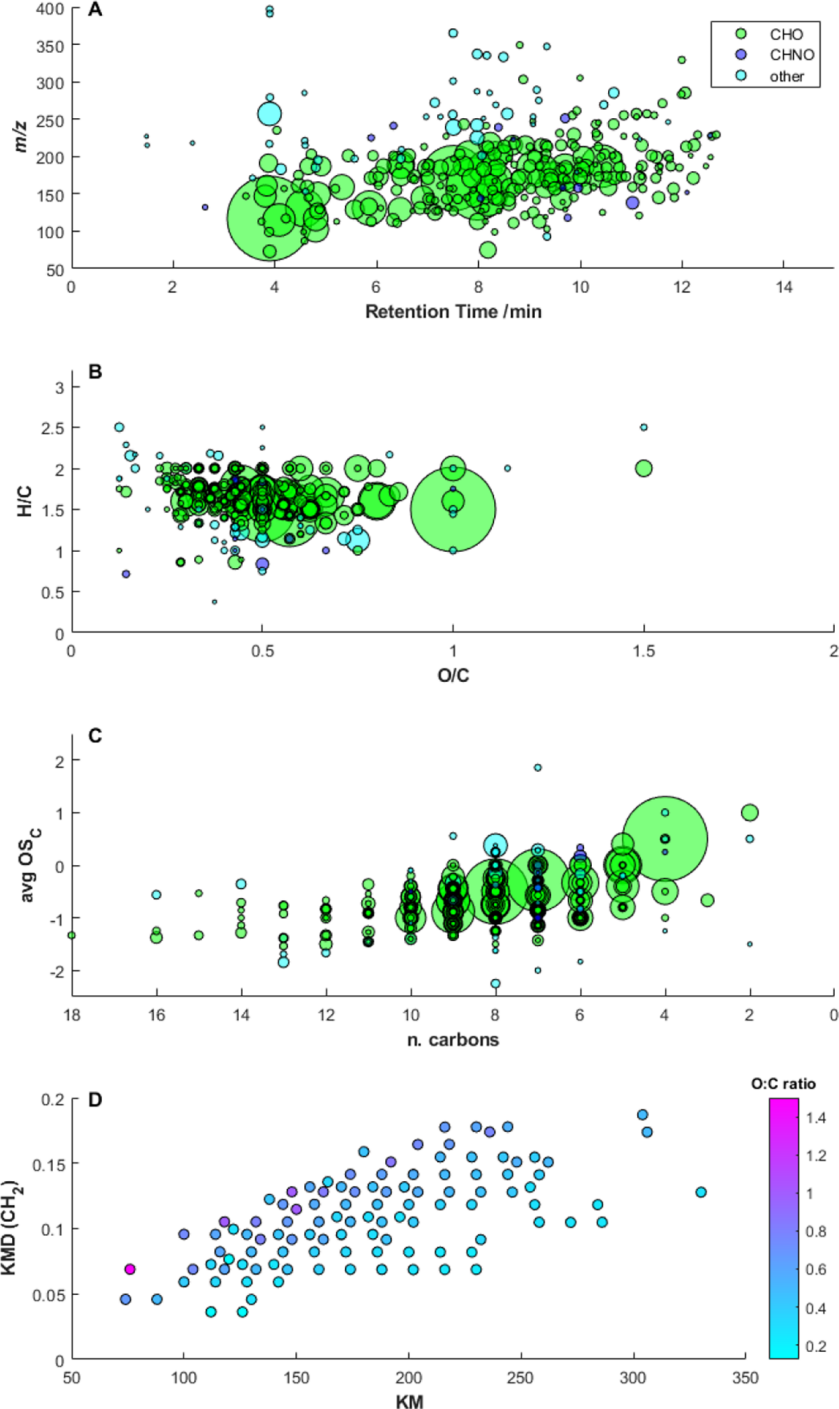
Overview of the NTS performed on a Belukha ice-core sample. (A) Mass-to-charge ratio as a function of the retention time. (B) Van Krevelen diagram. (C) Kroll diagram, where avg. OS_C_ is the average carbon oxidation state. The size of the circles is proportional to the integrated peak areas of the molecular ions. Green circles refer to CHO compounds, violet circles to CHNO compounds, light-blue circles to compounds defined as “other” (see text for details). (D) Kendrick mass defect plot where only CHO compounds are reported (CH_2_ as the unit base). Only compounds with an area >5 × 10^6^ are shown.

The untargeted analysis of a Belukha ice-core sample, dated back to 1934 CE, allowed the identification of 313 different compounds (Figure 2). The majority of the molecules, i.e., 80%, consisted of carbon (C), hydrogen (H), and oxygen (O). A total of 4% of the molecules also contained nitrogen (N), while the remaining 16% was labeled as other and is constituted either by molecules that contain other heteroatoms, such as chloride, fluoride, phosphorus, or by molecules identified at Level 5 (see below for a description of the identification levels). Most of the compounds have an *m/z* between 120 and 270 (Figure S5). Among the identified compounds, we focused on those that showed an area larger or equal than 3 × 10^8^ for further characterization. The sum of the intensities of these molecules (defined hereafter as suspects, *n* = 10) explains 35% of the intensities of all the compounds found in the sample. The confidence levels for compound identification in HRMS are defined by Levels in the Schymanski scale*.*^37^ Briefly, Level 5 is when the exact mass is available, Level 4 is when the unequivocal molecular formula is provided, Level 3 is when tentative candidate(s) is/are provided, Level 2 is when a probable structure is suggested (e.g., by library spectrum match or by diagnostic evidence), and Level 1 is when the probable structure is confirmed by a reference standard. Our attribution is based on the comparison of the MS/MS spectra with the mzCloud library (SI7) and of the reference standards, as well as from the comparison of the retention time (RT) of the suspects with the RT of the respective reference standards. When the difference between the RT is less than 0.1 min, the identity of the suspect is confirmed at Level 1.

Among the 10 compounds with the highest intensities (≥3 × 10^8^), four were identified at Level 4, two were identified at Level 2, while four were identified at Level 1 through the comparison with reference standards (Table 2). Those identified at Level 1 were typical dicarboxylic acids, such as succinic acid (C_4_H_6_O_4_) and glutaric acid (C_5_H_8_O_4_). Both are intermediate products of the photo-oxidation of azelaic acid (C_9_H_16_O_4_),^38^ which was also found in the sample. We also identified levulinic acid (C_5_H_8_O_3_), which is a γ-ketoacid. This compound is likely an oxidation product of 4-oxopentanal^39^ that in turn is an oxidation product of acyclic terpene compounds^40^ observed in both the gas and the particulate phase over forests.^41^ We also used authentic standards for the identification of C_6_H_10_O_4_ and C_5_H_8_O_4_, i.e., 3-methylglutaric acid and methylsuccinic acid, respectively. Due to the differences in the RT between standards and candidates (>0.1 min), these compounds were not identified at Level 1. However, due to MS/MS spectra similarities between standards and candidates, these compounds are likely to be isomers of 3-methylglutaric acid and methylsuccinic acid and can be identified at Level 2.

**Table 2:** Molecular Formula of the Compounds Showing the Largest Area (≥3 × 10^8^) Identified Following an Untargeted Screening Approach from a Belukha Ice-Core Sample^a^

molecular formula	name	theoretical *m/z*	Δmass (ppm)	RT (min)	area	mzCloud Best Match	frozen cartridge/unfrozen ratio	frozen vial/unfrozen ratio	confirmed by authentic standard
C_4_H_6_O_4_	succinic acid	117.01934	0.10	3.89	2.53 × 10^9^	87.2	1.147	1.192	YES (RT = 3.88)
C_8_H_12_O_4_	n.a.	171.06640	0.68	7.51	1.50 × 10^9^	n.a.	0.925	0.973	n.a.
C_7_H_10_O_4_	n.a.	157.05066	0.26	7.98	1.39 × 10^9^	n.a.	1.109	1.179	n.a.
C_9_H_16_O_4_	azelaic acid	187.09764	0.31	10.42	6.40 × 10^8^	92.8	0.996	1.104	YES (RT = 10.41)
C_5_H_8_O_4_	glutaric acid	131.03498	–0.01	4.58	5.00 × 10^8^	93.2	1.018	1.083	YES (RT = 4.53)
C_6_H_10_O_4_	isomer of 3-methylglutaric acid	145.05069	0.40	8.19	4.79 × 10^8^	87.5	1.006	1.097	NO (RT = 6.71)
C_5_H_8_O_4_	isomer of methylsuccinic acid	131.03497	–0.12	5.86	4.18 × 10^8^	94.6	1.032	1.206	NO (RT = 5.70)
C_5_H_8_O_3_	levulinic acid	115.04009	0.15	4.08	3.73 × 10^8^	89.8	1.118	0.878	YES (RT = 4.04)
C_10_H_16_O_3_	n.a.	183.10267	0.08	9.70	3.26 × 10^8^	n.a.	0.932	0.949	n.a.
C_9_H_14_O_5_	n.a.	201.07705	1.00	7.82	3.14 × 10^8^	n.a.	0.938	1.012	n.a.

a The authentic standards used for the identification of C_6_H_10_O_4_ and C_5_H_8_O_4_ were 3-methylglutaric acid and methylsuccinic acid, respectively. Due to the difference in the retention times (RT) between the standards and the suspects (>0.1 min), these compounds were not identified at Level 1, but as their respective isomers. n.a. refers to not applicable*.*

Extending our identification to all the compounds found in the sample and comparing the acquired MS/MS spectra with the ones available in the mzCloud online library, we were able to identify additional 99 compounds at Level 2, i.e., with at least one suggested probable structure. In Table S6, we report the compounds with an mzCloud Best Match ≥80 (*n* = 17). Among them, we found several secondary organic aerosol species, such as salicylic acid (C_7_H_6_O_3_) and adipic acid (C_6_H_10_O_4_), that together with glutaric acid can act as cloud condensation nuclei.^42^ Among the compounds detected at Level 2, we found *p*-hydroxybenzaldehyde (C_7_H_6_O_2_), which is a methoxyphenol released during biomass burning of Graminae together with *p*-hydroxybenzoic acid.^17^ Among the CHNO compounds, the most abundant was C_6_H_5_NO_3_ that we identified at Level 2 as *p*-nitrophenol. *p*-Nitrophenol is usually associated to biomass-burning activities,^28^ which is consistent with the occurrence in the same sample of *p*-hydroxybenzoic acid. This is an example on how merging a target and an untargeted approach can expand our knowledge about the chemical composition of an ice sample through the identification of other biomass-burning tracers that were not targeted at the beginning of our investigation.

The Van Krevelen diagram (Figure 2B) is often used for the graphical interpretation of high-resolution mass spectrometric data and is useful for the identification of specific molecular classes such as aromatics (H/C ratio < 1) or oxygenated organic aerosols (OOA) (1.2 < H/C < 1.9 and 0.3 < O/C < 1).^43,44^ Also, the Van Krevelen diagram is used to determine specific molecules’ oxidation pathways that involve the movement toward the right (higher O/C) and the bottom (lower H/C) part of the diagram itself when they get oxidized.^45^ In the analyzed ice-core sample, we observed that the majority of the identified CHO compounds lies within the area of OOA, while only few of them present aromatic features (including CHNO molecules), indicating that this methodology is particularly suitable for the former class of compounds.

The Kroll diagram (Figure 2C) introduces the average carbon oxidation state (avg OS_c_) that can be used to describe the oxidation state of the organic molecules. This metric, calculated as 2·O/C – H/C, always increases with the oxidation state of molecules and, when combined with carbon number (*n*_C_), restricts the composition of organic aerosol, providing information on the oxidative evolution of atmospheric compounds.^46^ Based on this diagram, the organic molecules can be divided into different classes according to their oxidation states and the number of carbon atoms, such as hydrocarbon-like organic aerosol, primary organic aerosols, or secondary organic aerosol. In our study, we found that most of the compounds occurred in the range −1 < avg OS_c_ < 0.5, which includes semi-volatile oxygenated organic aerosol (SV-OOA) and low-volatility oxygenated organic aerosol (LV-OOA), reflecting the atmospheric oxidative processing of primary organic aerosols promoted by oxidants such as OH radicals and ozone.^46,47^

The Kendrick mass defect (KMD) scale was developed by Edward Kendrick in 1963 as a powerful tool to detect and identify families of homologue compounds from complex high-resolution mass spectra.^48^ The corresponding Kendrick plot (Figure 2D) derives from mass spectra where *m/z* values are transformed into Kendrick masses (KM, on the *x*-axis), and the *y*-axis corresponds to the Kendrick mass defect (KMD). KM and KMD are calculated according to eq 1 and eq 2, where the nominal *M*_Kendrick reference_ and *M*_Kendrick reference_ are the nominal mass and the exact mass of the Kendrick reference used for the atomic mass unit definition, respectively.^49^ Here, we used as Kendrick reference CH_2_ (nominal *M*_Kendrick reference_ = 14.00000 and *M*_Kendrick reference_ = 14.01565). Following this approach, it is possible to identify homologues having the same KMD, and thus the same constitution of heteroatoms and number of rings plus double bonds, but a different number of CH_2_ groups.^50^
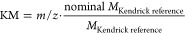
1
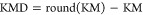
2

Among the different homologue series that we found in the Belukha sample, the most prominent one was the homologues of linear aliphatic dicarboxylic acids, C_2_O_4_H_2_(CH_2_)_*n*_ (2 ≤ *n* ≤ 7, KMD = 0.105). These compounds are of particular atmospheric relevance since they have the potential of acting as cloud condensation nuclei.^51^ Understanding their relative abundance is also a key to shed light on the atmospheric oxidative properties.^52^ We also found ω-hydroxy fatty acid homologues with general formula C_*n*_H_2*n*_O_3_ (2 ≤ *n* ≤ 13, KMD = 0.068), which can originate from higher plant waxes, soil microbes, or phytoplankton from surface ocean.^53^ Overall, the identification of homologue series is helpful for characterizing a larger amount of molecules when at least the identity of one homologue is known.

The methodology described in this paper represents an advancement of a previously published method for untargeted reconstructions of secondary organic aerosol tracers in ice cores.^28^ The two methods were compared through the analysis of two 30 mL aliquots from the same Belukha ice-core sample. One aliquot was treated and analyzed according to the work of Vogel et al.,^28^ while the other aliquot was analyzed following the method described here. The main differences between the two methods are reported in Table S7.

Following Vogel et al.’s methodology and using the same data analysis strategy, we identified 68 compounds. The majority of them is CHO (75%) followed by CHNO (7%) and CHOS (1%). The remaining 17% was labeled as “other”, and it refers either to molecules containing other heteroatoms (e.g., chlorine, fluorine, phosphorus, etc.) or to molecules that were identified at Level 5. Our new methodology captured 313 compounds, i.e., 4.6 times more molecules than the previous approach, with overall higher intensities. However, there are 19 compounds that were detected only with the Vogel et al.’s approach: 5 CHO compounds (C_5_H_6_O_4_, C_4_H_6_O_5_, C_8_H_6_O_4_, C_12_H_22_O_4_, and C_13_H_24_O_4_), 2 CHNO compounds (C_9_H_17_NO_3_ and C_12_H_23_NO_3_), 1 CHOS (C_4_H_8_O_3_S), and 11 compounds classified as “other”. In general, we conclude that the method presented here has the ability to capture a larger amount of CHO compounds with a higher sensitivity, although it did not overlap completely with the previous methodology (Figure S6). These discrepancies might arise from the differences in the SPE elution solutions between the two methods (i.e., the non-use of HCl in the new approach) and/or from the lower amount of formic acid used as a mobile phase additive that minimized signal suppression.

### 3.3 Re-freezing Samples

Due to external circumstances (e.g., instrument failure), it might be necessary to store ice and snow samples once they are already molten. In the following paragraphs, we discuss two sample storage strategies and we report the results of the freezing tests through both a targeted and an untargeted screening approach.

#### 3.3.1 Re-freezing Samples: Targeted Approach

The experiments carried out after spiking a Jungfraujoch snow sample at a concentration of ≈0.03 ng g^–1^ highlight that for some compounds, the accuracy and the reproducibility of the results are affected when the molten samples are frozen in glass vials (Figure S7). Significant sample losses between unfrozen (*n* = 4) and frozen (*n* = 4) aliquots were observed for syringic acid (*p*-value = 0.05), syringaldehyde (*p*-value <0.001), vanillin (*p*-value <0.001), and pinic acid (*p*-value = 0.02). In terms of concentrations, the observed decrease between unfrozen and frozen samples was 31% for syringic acid, 85% for syringaldehyde, 43% for vanillin, and 19% for pinic acid. For vanillic acid (*p*-value = 0.54) and *p*-hydroxybenzoic acid (*p*-value = 0.72), the difference between frozen and unfrozen aliquots was statistically insignificant. No significant changes were observed in the blanks between unfrozen (*n* = 2) and frozen (*n* = 2) samples. Differences between unfrozen (*n* = 4) and frozen (*n* = 4) aliquots were also observed, even though less pronounced, when the Jungfraujoch snow sample was spiked at a concentration of ≈0.1 ng g^–1^ (Figure S8). Significant losses were observed for vanillic acid (*p*-value = 0.01), pinic acid (*p*-value = 0.01), and *p*-hydroxybenzoic acid (*p*-value <0.01). However, the observed concentration decrease between frozen and unfrozen aliquots was less than 15%, consistent with the overall uncertainty of the method. For syringic acid (*p*-value = 0.79), vanillin (*p*-value = 0.15), and syringaldehyde (*p*-value = 0.45), no significant differences were observed between unfrozen and frozen samples. Blanks did not show any difference between the two aliquots (*n* = 2 for unfrozen samples and *n* = 2 for frozen samples).

To corroborate our findings, we performed a similar test using six different samples from the Colle Gnifetti ice core where each sample was divided in two different aliquots and spiked to reach a final added concentration of 0.03 ng g^–1^ (Figure S9). Results highlight that for syringic acid, vanillic acid, syringaldehyde, and vanillin, the frozen aliquots have different trends and concentration levels compared to the unfrozen ones of up to 74, 28, 38, and 63% lower compared to the unfrozen ones. For *p*-hydroxybenzoic acid and pinic acid, concentrations and trends were comparable, with the highest difference between frozen and unfrozen samples observed for *p*-hydroxybenzoic acid (i.e., 12%) that was anyway compatible with the analytical uncertainty of the method.

The heterogeneous behavior observed for the six targeted compounds among the different experiments indicate that the re-freezing of previously molten samples can be detrimental to the accuracy of the results especially at low concentrations. On these grounds and considering the expected low concentration levels in environmental ice and snow samples, we advise against re-freezing samples when syringic acid, vanillin, vanillic acid, and syringaldehyde are targeted and we encourage performing additional studies on the site-specific preservation of organics before re-freezing molten samples in glass vials.

To achieve a higher preservation of organic species, the freezing of previously loaded SPE cartridges was tested as an alternative of re-freezing samples in glass vials. This approach has been already used for targeted organic analyses from water samples, showing promising results.^54^ Similar to the previous experiments, we compared seven Colle Gnifetti samples, each of them was divided in two aliquots, both spiked to reach a final concentration of the targeted compounds of 0.03 ng g^–1^. One aliquot was immediately analyzed, while the other was loaded on the SPE cartridges and then frozen. The comparison between unfrozen samples and frozen cartridges shows good agreement (Figure S10). Furthermore, we tested the recovery from UPW samples prepared at 0.03 ng g^–1^ (*n* = 4), 0.1 ng g^–1^ (*n* = 4), and 1 ng g^–1^ (*n* = 4). We compared the results with the recoveries of the method (Figure S11) without finding any significant difference among the three concentration levels for all the targeted species, except for vanillin at 0.03 ng g^–1^, whose recovery was higher (70 ± 8%) in unfrozen samples than in the frozen samples (43 ± 7%). We conclude that freezing previously loaded SPE cartridges enhances the accuracy and the reproducibility of the results for the six targeted compounds compared to re-freezing previously molten samples.

Last, we also evaluated the preservation of the compounds when the samples are stored in the UHPLC-HRMS auto-sampler at 10 °C up to 48 h. A selected Colle Gnifetti unfrozen sample was analyzed multiple times after 24 and 48 h (three replicates for each day). Negligible losses (≤11%) were observed for vanillic acid, *p*-hydroxybenzoic acid, pinic acid, syringaldehyde, and vanillin after both 24 and 48 h. A 20% decrease was observed for syringic acid only after 48 h. Thus, in the case of instrumental failures, molten samples can be stored at 10 °C for at least 1 day without significant losses.

#### 3.3.2 Re-freezing Samples: Untargeted Screening Approach

The application of an NTS approach allowed a wider and more comprehensive evaluation of compounds’ preservation in ice samples. Their preservation was evaluated through the ratio between the areas of the compounds found in the frozen samples (i.e., frozen in glass vials or frozen cartridges) and the ones found in the unfrozen sample (e.g., a ratio of 1 means that a specific compound has the same area in both the unfrozen and in the frozen sample). The results show that most of the compounds were preserved independent of the chosen storage approach (Figure S12). More specifically, of 313 identified compounds, 88% (83%) were identified in the range of 0.8–1.2 ratios when the cartridge (glass vial) was frozen (Figure S13), respectively. Only 21 (20) compounds were found uniquely in the unfrozen sample compared to the frozen cartridge (frozen in glass vials).

Overall, these findings highlight that storing samples in frozen cartridges is a valuable approach also when an untargeted study is envisioned. The good agreement of the results between frozen cartridges and unfrozen samples further indicates the robustness of the method for untargeted studies since the majority of the identified compounds was found within the range of 0.9–1.1 (Figure S13). Even though we observed that the majority of the compounds is also preserved when the sample is frozen in glass vials, we recommend storing the samples in frozen cartridges since the agreement between the areas is stronger (Figure S13).

We conclude this section proposing a series of recommendations that can enhance the accuracy, the reproducibility, and the inter-laboratory comparability of the results when molten samples have to be stored before analysis:

(a) the analysis should be performed without refreezing the sample and within 24 h after sample melting to minimize the loss of different organic compounds, e.g., by adsorption to the glass surface of the vials, microbial degradation, and/or chemical reactions in the liquid-like ice grain boundaries;^55^
(b) if the analysis within 24 h is not possible and the samples need to be frozen (e.g., samples need to be transported, etc.), they should be stored in frozen SPE cartridges.

## 4 Conclusions

The presented methodology merges a targeted and a NTS approach to reconstruct past wildfire events from ice-core archives and, through an untargeted screening approach, allows the investigation of several hundreds of compounds. The method shows good sensitivity, recovery, and reproducibility for the simultaneous determination of pinic acid, syringic acid, syringaldehyde, vanillic acid, vanillin, and *p*-hydroxybenzoic acid. The application of an NTS approach allowed the identification of 313 different molecules from a single ice-core sample, which were mainly oxidation products of monoterpenes with *m/z* between 120 and 270. Among the most abundant molecules found in the Belukha ice-core sample, four were identified at Level 1, while additional 101 were identified at Level 2. Future ice-core reconstructions of wildfire and secondary organic tracers will benefit from this methodology to widen our knowledge on fire’s impact on atmospheric chemistry. In addition, the definition of a robust sample storage protocol will enhance the reproducibility when inter-laboratory studies are envisioned.

## Supporting Information Available

Chemicals and reagents, sample description and ice cutting, solid-phase extraction optimization, UHPLC-HRMS optimization, testing of different elution programs, freezing tests procedure, Compound Discoverer workflow and settings, MS/MS spectra of the identified compounds at Level 1/2 in the Belukha ice-core sample, comparison between the HPLC-(-)-ESI-MS limits of detection established in this study and other comparable methods, comparison between methodological limits of detection established in this study and the concentration ranges of the targeted compounds from different ice-core locations, evaluation of the method reproducibility for the targeted compounds from two aliquots of a selected ice-core sample, selection of the molecules identified at Level 2 after non-target screening analysis, main differences between the method presented in this work and the method presented by Vogel et al., pinic acid NMR spectrum, chromatographic separation of the targeted molecules, calibration curves of the targeted molecules, recovery of the targeted molecules at three different concentration levels, mass to charge (*m*/*z*) ratio distribution of the compounds after untargeted analysis, comparison in terms of number of identified compounds between the methodology presented in this work and Vogel et al.’ methodology, boxplots showing the differences between unfrozen and frozen aliquots at two different concentration levels in ultrapure water, comparison between frozen and unfrozen samples (*n* = 6) from the Colle Gnifetti ice core, comparison between unfrozen samples and samples frozen in SPE cartridges (*n* = 7) from the Colle Gnifetti ice core, recovery experiments performed spiking ultrapure water at three different concentration levels for not frozen samples and samples frozen in cartridges, ratio between the area retrieved from samples frozen in cartridges and the area of unfrozen samples after untargeted analysis (PDF)
