# Supplementary material for: Hybrid Targeted/Untargeted Screening Method for the Determination of Wildfire and Water-Soluble Organic Tracers in Ice Cores and Snow
Source: Anal Chem. 2023 Jul 18;95(30):11456–66. doi: 10.1021/acs.analchem.3c01852 (PMC10398623; doi:10.1021/acs.analchem.3c01852)
Supplement: Supplementary file 1 — ac3c01852_si_001.pdf [file ac3c01852_si_001.pdf]

SUPPLEMENTARY MATERIAL

Hybrid targeted/untargeted screening method for the determination of wildfire and water-soluble organic tracers in ice cores and snow

François Burgay[1,2], Daniil Salionov[3], Carla Jennifer Huber[1,2,4], Thomas Singer[1,2,4], Anja Eichler[1,2], Florian Ungeheuer[5], Alexander Vogel[5], Margit Schwikowski[1,2,4] & Saša Bjelić[3]*

[1]Laboratory of Environmental Chemistry (LUC), Paul Scherrer Institut, 5232 Villigen PSI, Switzerland

[2]Oeschger Centre for Climate Change Research, University of Bern, 3012, Bern, Switzerland

[3]Bioenergy and Catalysis Laboratory (LBK), Paul Scherrer Institut, 5232 Villigen PSI, Switzerland

[4]Department of Chemistry, Biochemistry and Pharmaceutical Sciences, University of Bern, 3012 Bern, Switzerland

[5]Institute for Atmospheric and Environmental Sciences (IAU), Goethe Universität, 60438 Frankfurt am Main, Germany

* Corresponding author: sasa.bjelic@psi.ch

Total pages: 34

Total number of tables: 7

Total number of figures: 13

SI1. Chemicals and reagents

Standard preparation: for the standard preparation we used syringic acid (≥ 95%, Sigma-Aldrich), vanillic acid (≥ 97.0%, Fluka), vanillin (ReagentPlus, 99%, Sigma-Aldrich), syringaldehyde (98%, Sigma-Aldrich) and *p*-hydroxybenzoic acid (ReagentPlus, ≥99%). For the identification of suspects we used levulinic acid (98%, Sigma-Aldrich), glutaric acid (99%, Sigma-Aldrich), succinic acid (>99.0%, TCI), methylsuccinic acid (>99.0%, TCI), 3-methylglutaric acid (>99.0%, TCI), azelaic acid (>98.0%, TCI). *p*-hydroxybenzoic acid-(phenyl-$^{13}C_6$) (99%, Sigma-Aldrich) and vanillin-(phenyl-$^{13}C_6$) (99%, Sigma-Aldrich) were the internal standards to monitor the solid phase extraction (SPE) and the instrumental performances, respectively. Pinic acid was synthetized as described in Steiner et al. (2018) and its NMR-spectrum is reported in Figure S1.

Solid phase extraction: ammonium hydroxide (25%, p.a. reag. ISO, Honeywell Fluka), hydrochloric acid (37%, Ph. Eur BP, NF), formic acid (≥ 98%, puriss. P.a., ACS reagent) and methanol (≥ 99.9%, for HPLC), all from Sigma Aldrich were used for solid phase extraction. Ultrapure Water (UPW) was produced in-house through a Sartorius Arium Pro water system (18.3 MΩ - 1 ppb TOC). SPE cartridges (Strong Anionic Exchange, MAX, 1 mL, 10 mg bed weight) were purchased from Waters. For the solid phase extraction, we used the Visiprep™ 12-port vacuum manifold (Supelco) equipped with PTFE disposable liners (Supelco). Disposable ≈ 50 cm-long PTFE transfer tubes (1/8'' OD, 1.59 mm ID, BGB) were used to load the SPE cartridges with the melted ice samples.

LC-MS analysis: acetonitrile (> 99.9%, Optima, LC/MS grade), methanol (> 99.9%, Optima, LC/MS grade) and water (Optima, LC/MS grade) were purchased from Fischer Chemical. Formic acid (98%, LC/MS grade) was purchased from Honeywell Fluka.

SI2. Sample description and ice cutting

To develop the method, several ice core sections and snow samples were used: n = 2 bulk snow sample from the Jungfraujoch station (3460 m.a.s.l. – 46°32.77 N, 7°58.66 E) and n = 15 ice-core samples from Colle Gnifetti (4450 m.a.s.l. - 45°55’45” N, 7°52’30” E). For the application of the method, n = 10 ice-core samples from Grand Combin (4123 m.a.s.l. - 45°56’16” N, 7°17’13” E) and n = 1 ice-core sample from the Belukha glacier (4062 m.a.s.l. - 49°48’26” N, 86°34’43” E) were used. All cores were sealed in polyethylene tubes in the field and stored in insulated boxes at -20°C. The snow samples were collected in 2 L pre-cleaned glass jars (§2.2 Labware decontamination procedure). All samples were shipped frozen to Paul Scherrer Institut (Switzerland) for analysis.

Ice cutting was performed in a cold room at -20°C using a modified band saw with a stainless steel blade and a polytetrafluoroethylene (PTFE) tabletop. All surfaces in contact with the core (i.e. the bandsaw, the tabletop and the saw guide) were cleaned at the beginning of the cutting day and after each core with acetone (Reag. Ph. Eur., VWR Chemicals). The operators wore heated gloves covered with polyethylene gloves that were changed after each core to avoid sample cross-contamination. To avoid any contamination from the ice-core drilling operations as well as from the ice core handling and storage, the outermost part (≈1 cm) of each ice-core section was removed. The decontaminated inner ice-core parts were used for organic analyses. For the Belukha and Colle Gnifetti ice cores (i.e. the method development cores), a section of 2.4 x 2.5 x 50 cm was cut for organic analyses, whereas for the Grand Combin ice core (i.e., the method application core) the section was 3.9 x 2 x 15 cm. The ice samples were stored in pre-cleaned polyethylene (PE) jars (method development cores) or in pre-cleaned 240 mL glass jars (method application core) and kept at -20°C (§2.3). The bulk snow samples from Jungfraujoch were aliquoted in 16 pre-cleaned 50 mL vials for re-freezing experiments (§SI5)

SI3. Solid-phase extraction optimization

The solid phase extraction procedure used in this method is similar to the one described by Vogel at al., 2019. However, due to the chromatographic column change (we used here an Organic Acid column, while Vogel et al., used a RP-MS column), we modified the SPE elution solutions. In Vogel et al., the elution was performed using a solution of 250 µL 0.5% HCl and 5% formic acid in methanol (hereafter, solution A), followed by a solution of 500 µL 5% formic acid in methanol (hereafter, solution B). Following the same approach, we observed a detrimental effect on the ionization performances, likely due to the presence of HCl residues after the evaporation and the non-suitability of the Organic Acid column with traces of a strong acid. Comparing the results of two extractions performed on three 0.1 ng g^{-1} UPW standard solutions, we observed that for the cartridges eluted using both solution A and B, the intensities of syringic acid, syringaldehyde, *p*-hydroxybenzoic acid and vanillic acid were 0.5, 0.8, 0.6 and 0.5 times the intensities obtained using 750 µL of solution B, only (not shown). For vanillin and pinic acid, the intensities were similar. In light of this, we eluted our cartridges using uniquely solution B.

SI4. UHPLC-HRMS optimization

Considering the acidic and polar properties of the compounds targeted in this study (i.e. methoxyphenols and secondary organic aerosol tracers), the chromatographic column used was the Acclaim^{TM} Organic Acid column, a silica-based reverse-phase column designed for the retention and following detection of hydrophilic aromatic and aliphatic acids. The gradient program was optimized in order to achieve the better sensitivity for the targeted compounds and to maximize the number of molecules that can be detected following an untargeted approach. After testing different gradients (Table S1), we opted for the 15 min gradient (#1 in Table S1) because: a) the sum of the targeted compounds intensities was higher, b) the number of identified compounds following an untargeted approach was the largest, and, c) the analysis time was the shortest.

Table S1 - Different elution programs (#1-#6) resulted in different target compound intensities and in different numbers of identifications. Here we considered only the compounds that showed an area higher than 1E7. To perform this study we used a sample from the Colle Gnifetti. The values were normalized for the maximum area among the different testing conditions for every single compound (e.g. 0.6 means that the intensity was 0.6 times the highest intensity). The finally applied program #1 is marked in bold. n.d. = not detected.

**Elution program #**	**1**	2	3	4	5	6
Analysis time /min	**15**	18	21	24	27	30
Gradient program	**0-12 min 8%-90% B**	0-15 min 8%-90% B	0-18 min 8%-90% B	0-21 min 8%-90% B	0-24 min 8%-90% B	0-27 min 8%-90% B
**Identifications #**	**191**	165	67	109	89	79
**Syringic acid**	**1**	0.7	0.6	n.d.	n.d.	n.d.
**Vanillic acid**	**1**	0.7	0.6	0.2	n.d.	n.d.
**Vanillin**	**1**	0.6	0.6	n.d.	n.d.	n.d.
**Syringaldehyde**	**1**	0.7	0.6	n.d.	n.d.	n.d.
***p*-hydroxybenzoic acid**	**1**	0.7	0.6	n.d.	0.2	n.d.
**Pinic acid**	**1**	0.8	0.4	0.4	0.4	0.3
**Sum**	**6**	4.2	3.4	0.6	0.5	0.3

The instrumental method was optimized after testing eluents with different modifier concentrations, with and without post-column addition of NH_4OH (Table S2). We did not observe an overall improvement in sensitivity when the post-column addition of aqueous ammonia was included, probably due to the formation of salt adducts that had a detrimental effect on the ionization efficiency. We found that the overall optimal eluent modifier concentration for the six target species was 0.001% formic acid. However, we opted for the 0.01% concentration that improved the sensitivity up to 7 times compared to previous methods (0.2% formic acid) (Vogel et al., 2019), and it was only 13% lower than the 0.001% option. This choice was to ensure a high sensitivity also for pinic acid and, consequently, for other similar aliphatic carboxylic acids that can be detected following a NTS approach.

Table S2 - Average normalized integrated area for different concentrations of formic acid (FA) added as a modifier in eluent A (1% ACN, 1% MeOH in UPW). The post-column addition of acqueous NH_4OH was also tested at a concentration of 300 mM and flow of 8-μL min^{-1}. The values were normalized for the maximum area obtained among the different testing conditions for every single compound. The experiment was performed using a standard solution prepared in UPW at 1 ng g^{-1}. The finally applied eluent modifier concentration is marked in bold. n.d. = not detected.

	FA 0.001%	FA 0.001% NH_3	**FA 0.01%**	FA 0.01% NH_3	FA 0.2%
**Syringic acid**	0.8	0.5	**1**	0.4	n.d.
**Vanillic acid**	1	0.5	**0.6**	0.3	0.2
**Vanillin**	1	0.8	**0.4**	0.2	0.02
**Syringaldehyde**	1	0.6	**0.5**	0.3	0.05
***p*-hydroxybenzoic acid**	0.6	0.5	**1**	0.4	0.2
**Pinic acid**	0.6	0.3	**1**	0.2	0.2
**Sum**	5.1	3.2	**4.4**	1.8	0.7

SI5. Freezing tests

Besides applying the method to investigate the chemical composition of snow and ice core samples, we investigated the preservation of organic molecules when two different sample storage approaches are used: a) re-freezing of a previously molten sample and b) freezing of previously loaded SPE cartridges. With our study we provide evidences on the preservation of the chemical species following both a target approach (§3.3.1 main text) and a NTS approach (§3.3.2 main text).

Re-freezing samples in glass vials *(target approach)*

To evaluate whether the six target compounds were preserved after the refreezing of previously molten ice samples, we carried out two sets of experiments with snow collected from Jungfraujoch and spiked at concentrations of ≈ 0.03 ng g^{-1} (n = 8) and ≈ 0.1 ng g^{-1} (n = 8), respectively. For each concentration, four samples were extracted and analysed the same day (unfrozen samples), while the other four samples were frozen at -20°C for 24 hours, then again molten, extracted and analysed (frozen samples). The experiments are summarized as follows:

a) 400 g of snow collected at the Jungfraujoch station (3460 m.a.s.l. – 46°32.77 N, 7°58.66 E) were molten and spiked with the targeted compounds to reach a final spiked concentration of 0.03 ng g^{-1}. The sample was divided into eight aliquots: four (and one blank) were extracted and analysed the same day, while the other four (and one blank) were frozen for 24 h (-20°C), then molten, extracted and analysed.
b) as for a), but the spiked concentration was 0.1 ng g^{-1} ;
c) 2x30 mL sample aliquots from the Colle Gnifetti ice core were spiked to reach a final added concentration of ≈ 0.03 ng g^{-1}. One aliquot was immediately extracted and analysed, while the other was kept frozen for seven days (-20°C), then extracted and analysed. This procedure was repeated for six different samples. Six blanks were also collected and analysed.

Re-freezing samples in SPE cartridges *(target approach)*

To explore alternative strategies to sample re-freezing in glass vials, we also investigated the compound's stability once loaded on a SPE cartridge. We performed different experiments:

a) We evaluated the sample recovery from frozen cartridges in UPW at three different concentrations (i.e. 0.03 ng g^{-1} [n=3], 0.1 ng g^{-1} [n=4] and 1 ng g^{-1} [n=4]) and compared the results with those obtained from unfrozen cartridges.

b) 2x30 mL sample aliquots from Colle Gnifetti ice core were spiked to reach a final added concentration of ≈ 0.03 ng g^{-1}. Both aliquots were loaded on two different SPE cartridges. One cartridge (and one blank) was eluted and analysed the same day. The other cartridge (and an additional blank) was dried under vacuum for 5 minutes, then wrapped into two aluminium foils and stored for 7 days at -20°C. Before elution, the cartridge was thawed at room temperature for ≈ 30 minutes under Class-1000 laminar flow hood, then eluted and analysed. This procedure was repeated for seven different samples. Six blanks were also collected and analysed.

Re-freezing samples in glass vials and SPE cartridges *(untargeted screening approach)*

Finally, to extend our analysis to a wider set of compounds, we performed a NTS on three 30 mL aliquots from a Belukha ice-core sample. One aliquot was immediately extracted and analyzed. The second aliquot was frozen into a 50 mL glass vial for seven days at -20°C, then extracted with SPE, eluted and analyzed. The third aliquot was loaded on a SPE cartridge, frozen for seven days at -20°C, then eluted and analyzed. An UPW blank was prepared in parallel for each aliquot and treated the same as the sample.

SI6. Compound Discoverer Workflow and Settings

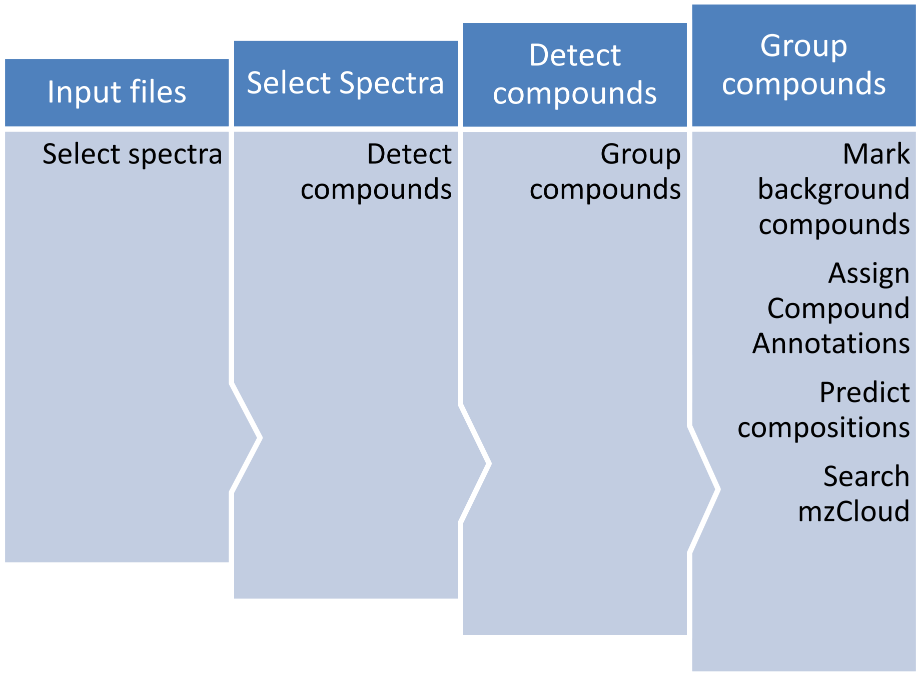

Processing node 51: Detect Compounds

----

1. General Settings:

- Mass Tolerance [ppm]: 2.5 ppm
- Min. Peak Intensity: 1000000
- Min. # Scans per Peak: 5
- Use Most Intense Isotope Only: True

2. Trace Detection:

- Max. Number of Gaps to Correct: 2
- Min. Number of Adjacent Non-Zeros: 2

3. Peak Detection:

- Chromatographic S/N Threshold: 3
- Remove Baseline: False
- Gap Ratio Threshold: 0.35

- Max. Peak Width [min]: 1

- Min. Relative Valley Depth: 0.1

4. Isotope Pattern Detection:

- Group Isotopes for: Br; Cl

- Use Peak Quality for Isotope Grouping: True

- Filter out Features with Bad Peaks Only: True

- Zig-Zag Index Threshold: 0.2

- Jaggedness Threshold: 0.4

- Modality Threshold: 0.9

- Remove Potentially False Positive Isotopes: False

5. Compound Detection:

- Ions:

    [2M+ACN+H]+1

    [2M+ACN+Na]+1

    [2M+FA-H]-1

    [2M+H]+1

    [2M+K]+1

    [2M+Na]+1

    [2M+NH4]+1

    [2M-H]-1

    [2M-H+HAc]-1

    [M+2H]+2

    [M+3H]+3

    [M+ACN+2H]+2

    [M+ACN+H]+1

    [M+ACN+Na]+1

    [M+Cl]-1

    [M+DMSO+H]+1

[M+FA-H]-1

[M+H]+1

[M+H+K]+2

[M+H+MeOH]+1

[M+H+Na]+2

[M+H+NH4]+2

[M+H-H2O]+1

[M+H-NH3]+1

[M+K]+1

[M+Na]+1

[M+NH4]+1

[M-2H]-2

[M-2H+K]-1

[M-H]-1

[M-H+HAc]-1

[M-H+TFA]-1

[M-H-H2O]-1

- Base Ions: [M+H]+1; [M+NH4]+1; [M-H]-1

- Remove Singlets: True

Processing node 25: Group Compounds

----

1. General Settings:

- Mass Tolerance: 2.5 ppm

- RT Tolerance [min]: 0.1

- Align Peaks: False

- Preferred Ions: [M+H]+1; [M+NH4]+1; [M-H]-1

- Area Integration: Most Common Ion

2. Peak Rating Contributions:

- Area Contribution: 3

- CV Contribution: 10

- FWHM to Base Contribution: 5

- Jaggedness Contribution: 5

- Modality Contribution: 5

- Zig-Zag Index Contribution: 5

3. Peak Rating Filter:

- Peak Rating Threshold: 5

- Number of Files: 2

Processing node 43: Mark Background Compounds

----

1. General Settings:

- Max. Sample/Blank: 5

- Max. Blank/Sample: 0

- Hide Background: True

Processing node 40: Assign Compound Annotations

----

1. General Settings:

- Mass Tolerance: 2.5 ppm

2. Data Sources:

- Data Source #1: mzCloud Search

- Data Source #2: mzVault Search

- Data Source #3: MassList Search

- Data Source #4: Predicted Compositions

- Data Source #5: ChemSpider Search

- Data Source #6: (not specified)

- Data Source #7: (not specified)

3. Scoring Rules:

- Use mzLogic: True
- Use Spectral Distance: True
- SFit Threshold: 20
- SFit Range: 20

4. Reprocessing:

- Clear Names: False

Processing node 37: Predict Compositions

---

1. Prediction Settings:

- Mass Tolerance: 2.5 ppm
- Min. Element Counts: C H
- Max. Element Counts: C90 H190 Br3 Cl8 F18 N10 O18 P3 S5
- Min. RDBE: -2
- Max. RDBE: 40
- Min. H/C: 0.1
- Max. H/C: 3.5
- Max. # Candidates: 10
- Max. # Internal Candidates: 500

2. Pattern Matching:

- Intensity Tolerance [%]: 30
- Intensity Threshold [%]: 0.1
- S/N Threshold: 3
- Min. Spectral Fit [%]: 30
- Min. Pattern Cov. [%]: 80
- Use Dynamic Recalibration: True

3. Fragments Matching:

- Use Fragments Matching: True
- Mass Tolerance: 5 ppm
- S/N Threshold: 3

Processing node 52: Search mzCloud

----

1. General Settings:

- Compound Classes: All
- Precursor Mass Tolerance: 10 ppm
- FT Fragment Mass Tolerance: 10 ppm
- IT Fragment Mass Tolerance: 0.4 Da
- Library: Autoprocessed; Reference
- Post Processing: Recalibrated
- Max. # Results: 10
- Annotate Matching Fragments: False
- Search MSn Tree: False

2. DDA Search:

- Identity Search: HighChem HighRes
- Match Activation Type: True
- Match Activation Energy: Match with Tolerance
- Activation Energy Tolerance: 20
- Apply Intensity Threshold: True
- Similarity Search: None
- Match Factor Threshold: 60

3. DIA Search:

- Use DIA Scans for Search: False

- Max. Isolation Width [Da]: 500
- Match Activation Type: False
- Match Activation Energy: Any
- Activation Energy Tolerance: 100
- Apply Intensity Threshold: False
- Match Factor Threshold: 20

SI7. MS/MS spectra of the identified compounds from the Belukha ice core at Level 1/Level 2 Confidence Level (*Schymanski Scale*). Reference spectrum is at bottom.

	RT sample	RT reference	mzCloud BestMatch
**Succinic acid**	**3.89**	**3.88**	**87.2**

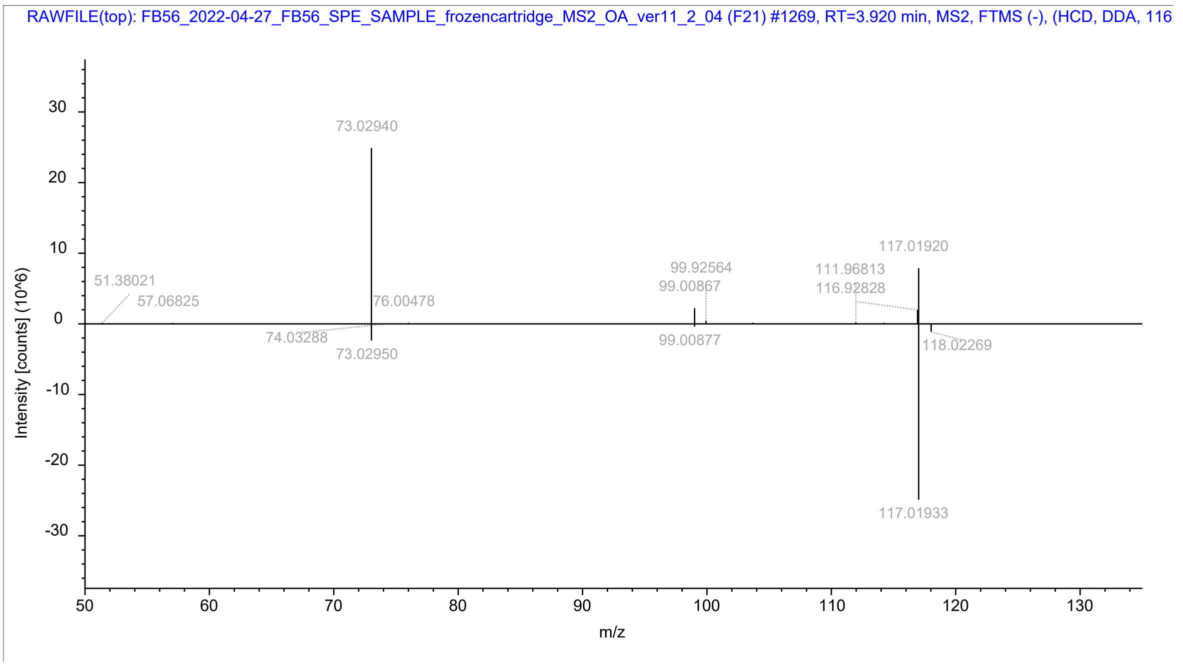

	RT sample	RT reference	mzCloud BestMatch
**Azelaic acid**	**10.42**	**10.41**	**92.8**

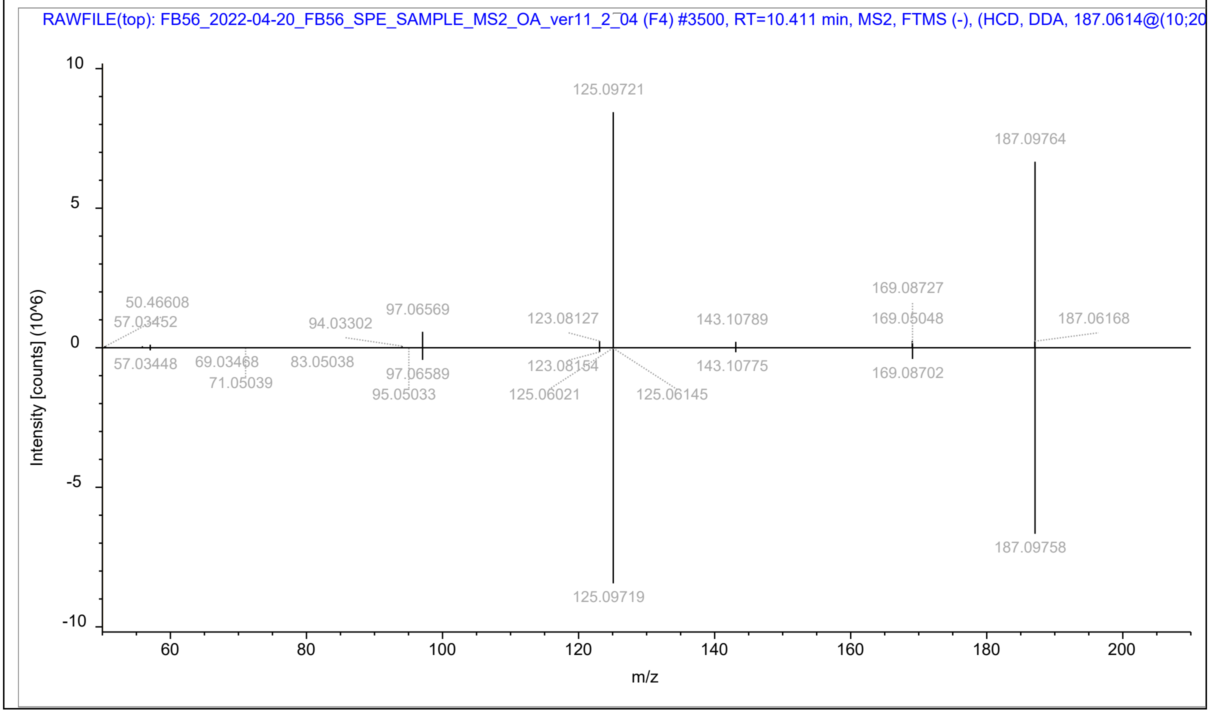

	RT sample	RT reference	mzCloud BestMatch
**Glutaric acid**	**4.58**	**4.53**	**93.2**

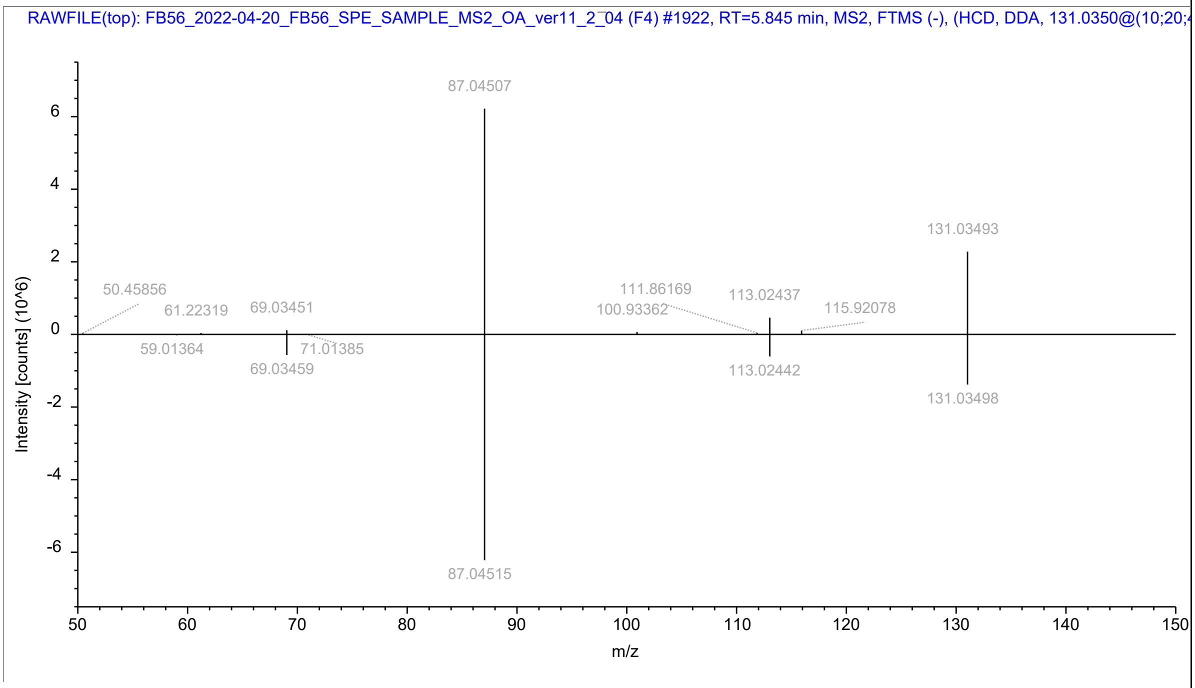

	RT sample	RT reference	mzCloud BestMatch
**Levulinic acid**	**4.08**	**4.04**	**89.8**

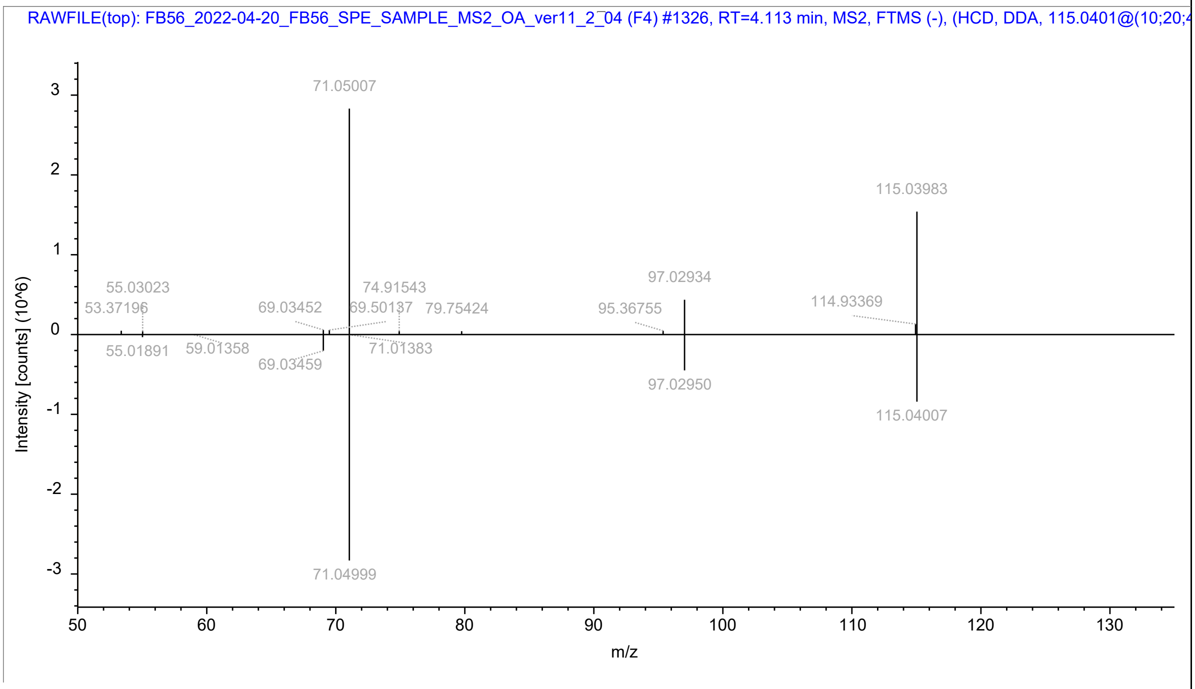

	RT sample	RT reference	mzCloud BestMatch
**p-nitrophenol**	**11.03**	**n.a.**	**97.1**

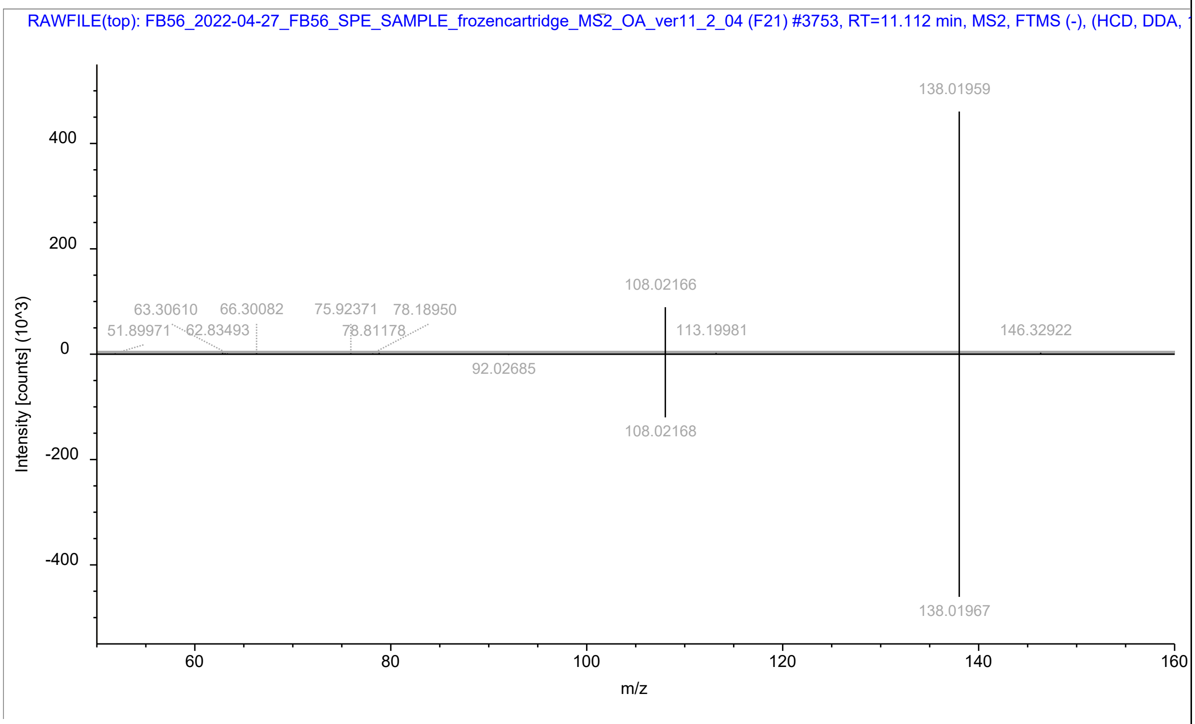

	RT sample	RT reference (methylsuccinic acid)	mzCloud BestMatch
Isomer of methylsuccinic acid	**5.86**	**5.70**	**94.6**

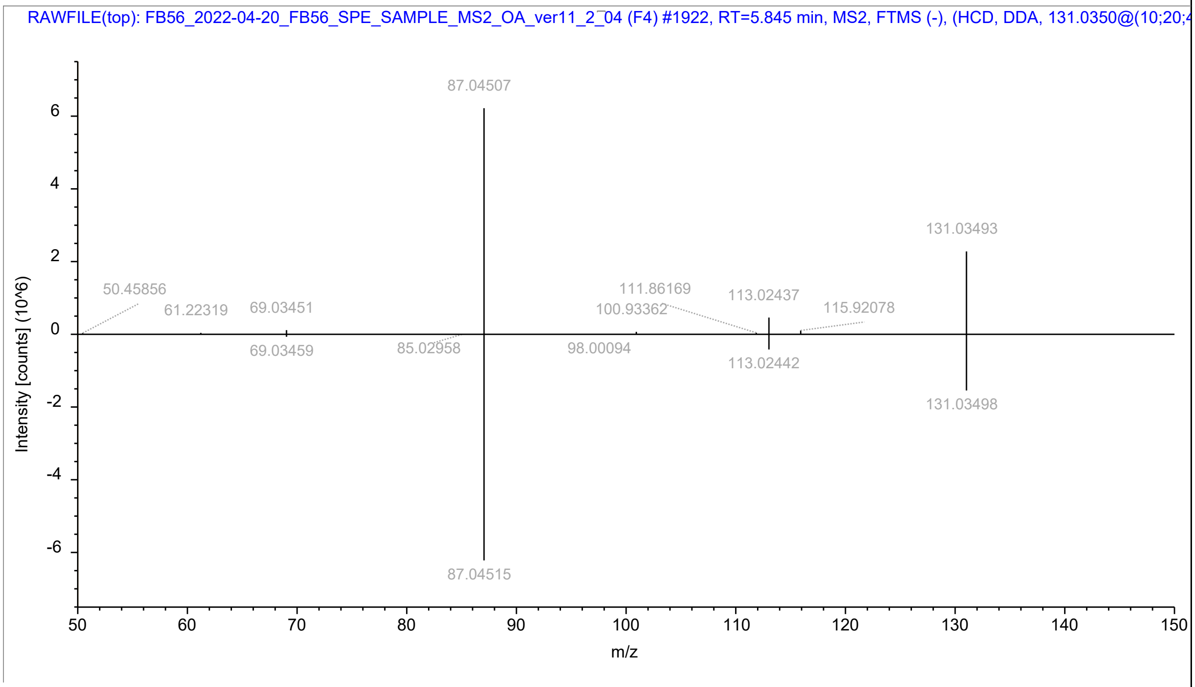

	RT sample	RT reference (*3-methylglutaric acid*)	mzCloud BestMatch
Isomer of 3-methylglutaric acid	**8.19**	**6.71**	**87.5**

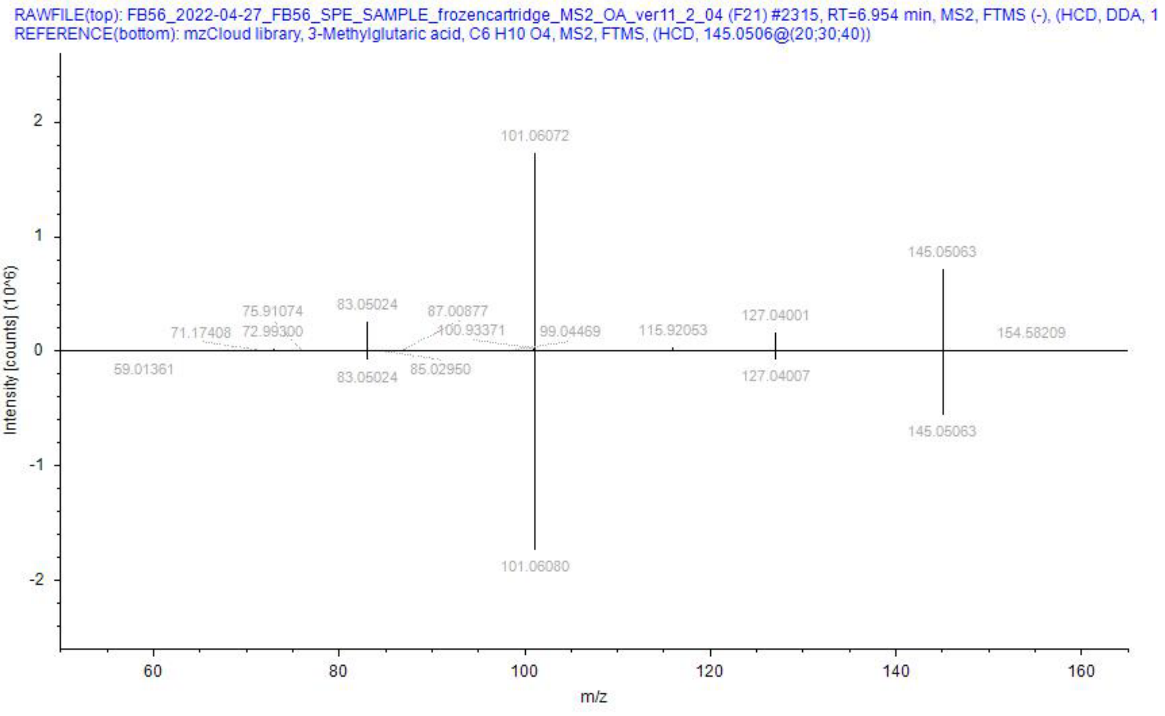

Additional Tables & Figures

Table S3 - Comparison between the HPLC-(-)ESI-MS method established in this study and other comparable methods in terms of instrumental LoD (pg) and MDL (ng g^{-1}). Data from Muller-Tautges were calculated considering the injection volume and the amount of sample used for pre-concentration (80 mL). Grieman et al., (2017) refers to an IC-(-)ESI-MS method. NA = not available.

	**This study**		**Grieman et al. 2015**		**Müller-Tautges et al. 2016**		**Grieman et al. 2017**	
	**LOD**	**MDL**	LOD	MDL	LOD	MDL	LOD	MDL
**Syringic acid**	**12**	**0.005**	NA	NA	NA	NA	NA	0.09
**Vanillic acid**	**7**	**0.012**	7.7	NA	16.8	NA	NA	0.01
**Vanillin**	**4**	**0.007**	NA	NA	NA	NA	NA	NA
**Syringaldehyde**	**10**	**0.003**	NA	NA	NA	NA	NA	NA
***p*-hydroxybenzoic acid**	**2.5**	**0.007**	NA	NA	7.2	NA	NA	0.012
**Pinic acid**	**3**	**0.010**	NA	NA	0.2	NA	NA	NA
**Analysis time /min**	15		4		7		14	
**Flow rate / µL min^{-1}**	200		200		500		200	
**Injection volume /µL**	20		100		10		1000	

Table S4 - Comparison between the methodological limit of detection of this method with the concentration range of the six target compounds in ice and snow samples from different locations. MDL = methodological limit of detection (this study). ND = not detected, below methodological LoD of the cited study. NA = not available.

**Compound**	**MDL /ng g^{-1}**	**Alps[a,b] /ng g^{-1}**	**Greenland[c] /ng g^{-1}**	**Kamchatka[d] /ng g^{-1}**	**Tibetan plateau[e] /ng g^{-1}**	**Svalbard[f] /ng g^{-1}**	**Russian Arctic[g,h] /ng g^{-1}**
**Syringic acid**	0.005	ND	ND	NA	NA	NA	ND
**Vanillic acid**	0.012	0.021-0.5	≈ 0.08	0.005-0.127	0.015-0.361	0.003-0.2	0.01-1.5
**Vanillin**	0.007	NA	NA	NA	NA	NA	NA
**Syringaldehyde**	0.003	NA	NA	NA	NA	NA	NA
***p*-hydroxybenzoic acid**	0.007	0.009-0.151	NA	0.005 -1.741	0.033 - 0.568	0.006-0.07	0.05-1.5
**Pinic acid**	0.010	0.0003-0.92	NA	NA	NA	NA	NA

[a] (Müller-Tautges et al., 2016)
[b] (Barbaro et al., 2022)
[c] (Mcconnell et al., 2007)
[d] (Kawamura et al., 2012)
[e] (Gao et al., 2015)
[f] (Grieman et al., 2018)
[g] (Grieman et al., 2017)
[h] (Grieman et al., 2015)

Table S5 - Evaluation of the reproducibility from a selected Colle Gnifetti ice core sample (named 169) divided in two 30 mL aliquots (169A and 169B). The aliquots were spiked with the six targeted compounds to achieve a final added concentration of ≈ 0.03 ng g^{-1}.

	Sample_169A /ng g^{-1}	Sample_169B /ng g^{-1}	**Average /ng g^{-1}**	Std. Dev	RSD%
**Syringic acid**	0.031	0.029	**0.030**	0.001	3.9%
**Vanillic acid**	0.028	0.025	**0.026**	0.001	4.1%
**Vanillin**	0.02	0.01	**0.02**	0.01	32%
**Syringaldehyde**	0.0453	0.0463	**0.0458**	0.0005	1.0%
***p*-hydroxybenzoic acid**	0.0449	0.0455	**0.0452**	0.0003	0.6%
**Pinic acid**	0.27	0.28	**0.27**	0.01	1.9%

Table S6 – A selection of the molecules identified at Level 2 of the *Schymanski's scale*. Only those species showing an mzCloud Match ≥ 80 (n=17) are reported (i.e. four CHNO molecules and thirteen CHO molecules). Molecules are ranked accordingly to their area intensity.

Suspect name	Formula	*m/z*	Δmass /ppm	RT /min	Area	#mzCloud results	mzCloud Best Match	frozencartridge /unfrozen ratio	frozenvial/unfrozen ratio
Suberic acid	$C_8H_{14}O_4$	173.0819	-0.16	9.47	3.3E8	1	91.8	1.004	1.082
4-hydroxybutyric acid	$C_4H_8O_3$	103.0401	-0.04	4.80	2.6E8	2	83.8	1.080	0.948
Isomer of 3-methylglutaric acid	$C_6H_{10}O_4$	145.0507	0.27	6.90	2.1E8	4	93.3	0.988	1.083
3,3-dimethylglutaric acid	$C_7H_{12}O_4$	159.0663	0.29	9.32	1.8E8	4	94.5	1.021	1.072
1-(Carboxymethyl)cyclohexanecarboxylic acid	$C_9H_{14}O_4$	185.082	0.38	10.10	1.1E8	1	92.9	1.035	1.118
NP-022068	$C_8H_{12}O_5$	187.0613	0.39	6.13	8.1E7	1	80.5	0.917	0.999
Salicylic acid	$C_7H_6O_3$	137.0244	0.17	8.59	7.7E7	3	93.2	0.934	1.101
p-nitrophenol	$C_6H_5NO_3$	138.0197	0.27	11.03	5.7E7	6	97.1	0.838	0.888
12-hydroxydodecanoic acid	$C_{12}H_{24}O_3$	215.1654	0.65	12.24	5.7E7	1	81.9	0.912	1.018
N-isovalerylglycine	$C_7H_{13}NO_3$	158.0823	0.06	9.96	3.3E7	3	82.4	0.976	1.085
Adipic acid	$C_6H_{10}O_4$	145.0507	0.22	4.66	2.8E7	3	84.2	1.044	1.069
2-hydroxycinnamic acid	$C_9H_8O_3$	163.0402	0.53	9.31	2.3E7	4	87.1	1.143	1.134
Camphanic acid	$C_{10}H_{14}O_4$	197.0821	0.67	9.31	2.2E7	1	86.0	1.041	1.009

…continue

10-hydroxy-2-decenoic acid	$C_{10}H_{18}O_3$	185.1184	0.38	11.95	2.1E7	1	83.0	0.814	0.837
4-acetamidobutanoic acid	$C_6H_{11}NO_3$	144.0667	0.29	8.04	2.0E7	2	82.6	1.014	1.044
Aspartic acid	$C_4H_7NO_4$	132.0302	0.05	2.63	1.8E7	1	84.5	1.063	1.062
p-hydroxybenzaldehyde	$C_7H_6O_2$	121.0295	-0.02	11.16	1.8E7	2	97.6	0.876	0.897

Table S7 – Main differences between the method presented in this work and the methodpresented in Vogel et al., 2019. NA = not applied. MeOH = methanol, FA = formic acid, ACN = acetonitrile.

	**This work**	**Vogel et al., 2019**
	**SPE procedure**	
**Sample volume**	30 mL	60 mL
**Decontamination solution**	0.16M HCl in MeOH	NA
**Counter-ion solution**	0.5M FA in water	NA
**Elution solutions**	5% FA in MeOH	0.5% HCl and 5% FA in MeOH<br>5% FA in MeOH
	**Instrument optimizations**	
**UHPLC column**	Acclaim™ Organic Acid column	Accucore™ RP-MS
**Eluent A**	0.01% FA, 1% MeOH, 1% ACN	0.2% FA, 1% MeOH, 1% ACN
**Eluent B**	MeOH	MeOH
**Gradient**	0-12 min linear increase from 8% to 90% B<br>12-15 min 90% B	0-2 min 1% B<br>2-12 linear increase to 99%B<br>12-16 99%B
**Flow rate**	200 µL/min	400 µL/min
	**Mass-spectrometer parameters**	
**Scan range *(m/z)***	70-1000	70-1000
**Sheath gas**	35	50
**Auxiliary gas**	10	13
**Capillary voltage**	2.5 kV	3.4 kV

Figure S1 - Pinic acid was synthesized according to previously published procedure (Steimer et al., 2018). Its NMR spectrum is shown below. The signal at 3.78 ppm is associated to dioxane. Afterwards the product was further dried. The pinic acid measured exact mass of $[M-H]^-$ was 185.0820 (Δ<1 ppm).

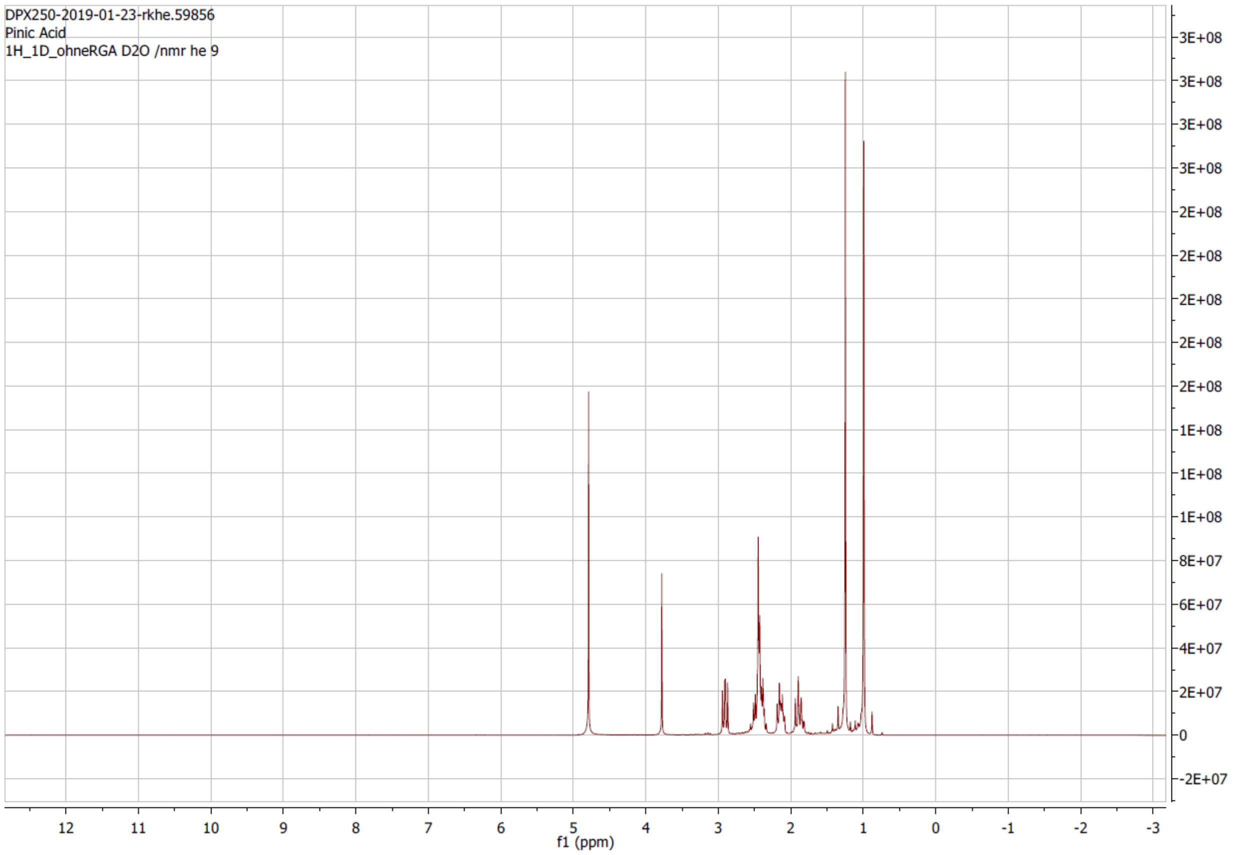

Figure S2 – Chromatographic separation of the targeted species at a concentration of 10 ng g^{-1}.

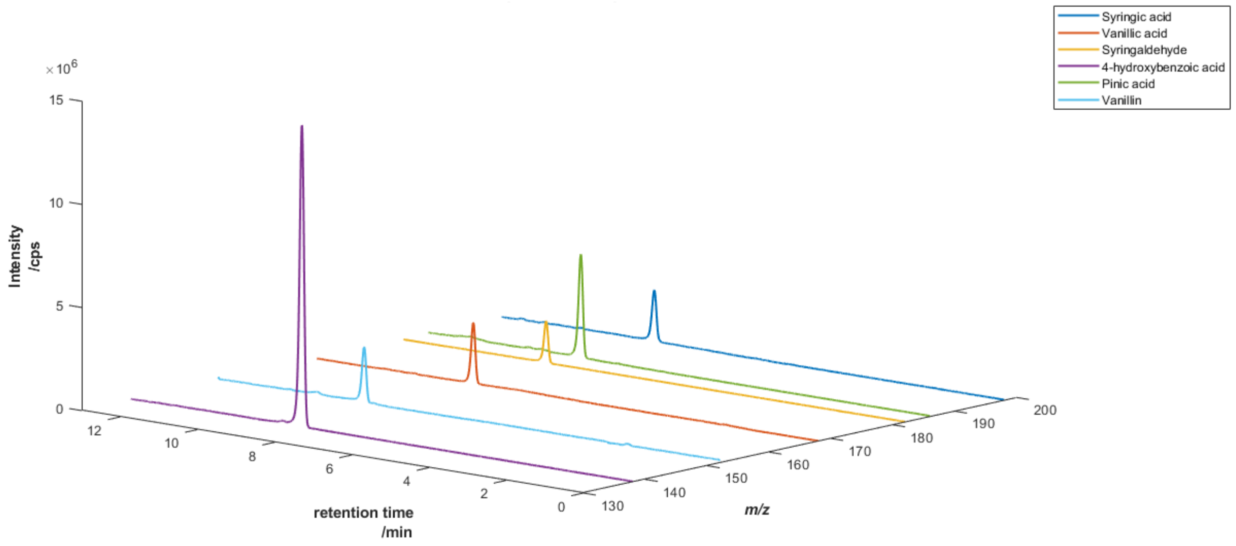

Figure S3 – Calibration curves for the six targeted species over the range 0.5-15 ng g^{-1}. Data are presented as area ratio (i.e. the ratio between the area of the targeted species and the area of the internal standard) vs concentration ratio (i.e. the ratio between the concentration of the targeted species and the concentration of the internal standard). The internal standard is vanillin-(phenyl-$^{13}C_6$). More details are reported in the main text.

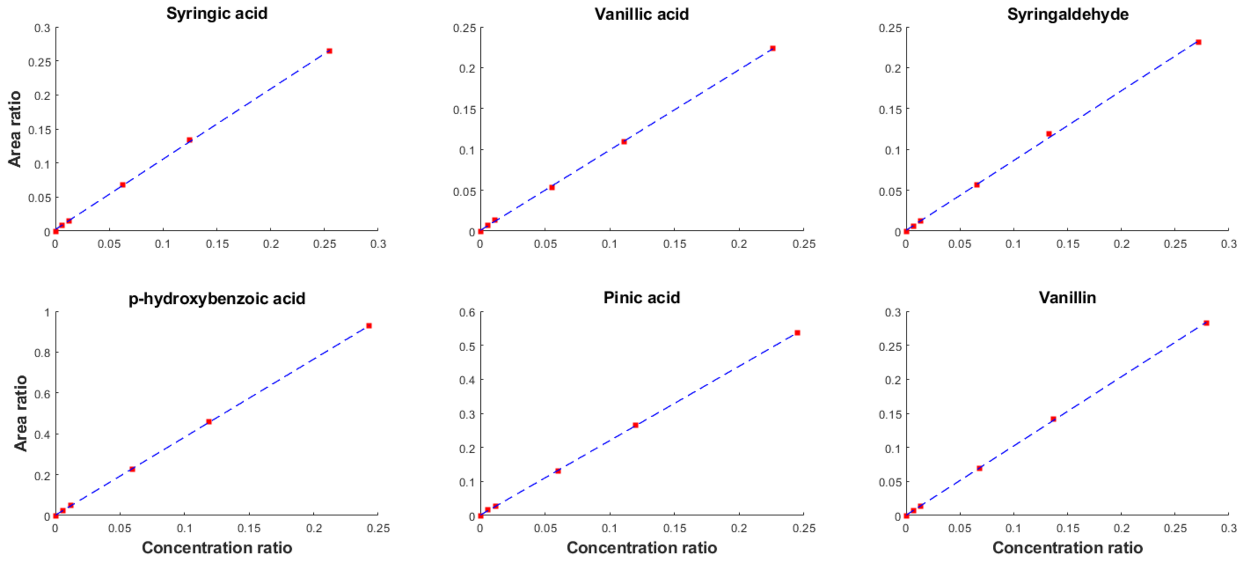

Figure S4 – Details on the recovery experiments performed on fresh UPW samples prepared at 0.03 (blue bar, n = 3), 0.1 (orange bar, n = 4) and 1 ng g^{-1} (yellow bar, n = 4).

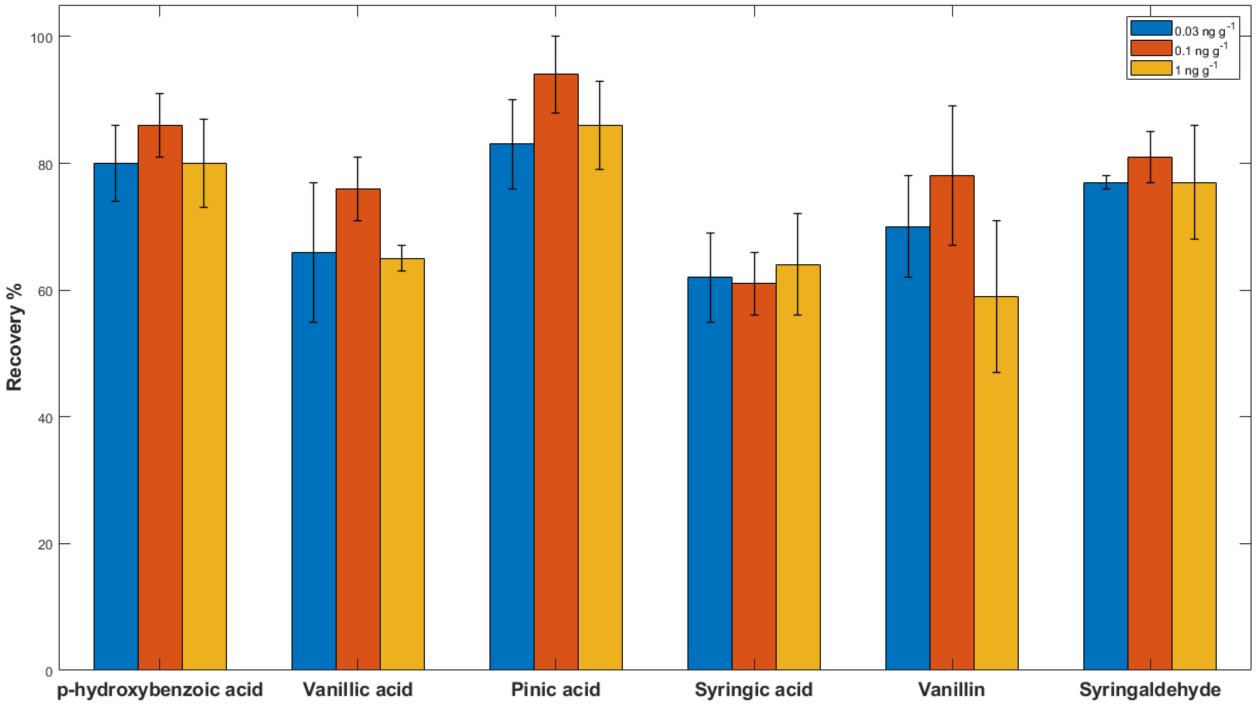

Figure S5 – Mass to charge (*m/z*) ratio distribution from the untargeted analysis of a Belukha ice core sample. The majority of the compounds detected (76%) has an *m/z* between 120 and 270.

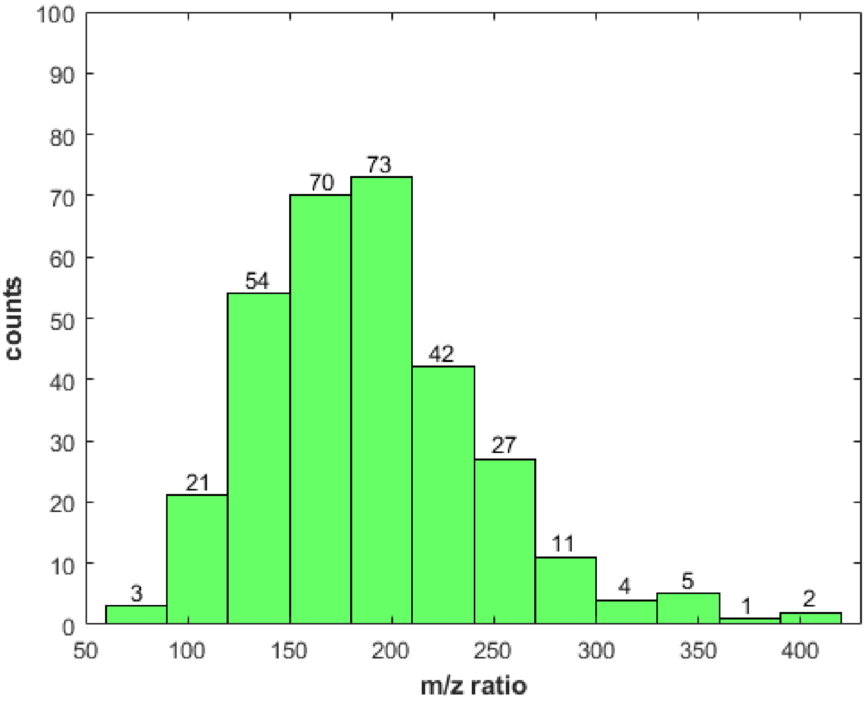

Figure S6 – Comparison between a non-target screening from a Belukha ice core sample using the methodology presented in this work (panel A) and Vogel et al. (2019) methodology (panel B). Both samples were extracted and immediately analyzed. The total amount of detected compounds increased from 68 (panel B), to 313 (panel A). Only compounds with area >5E6 and *m/z* between 75 and 400 are shown. The size of the circle represents the relative area intensities. More details are in the main text.

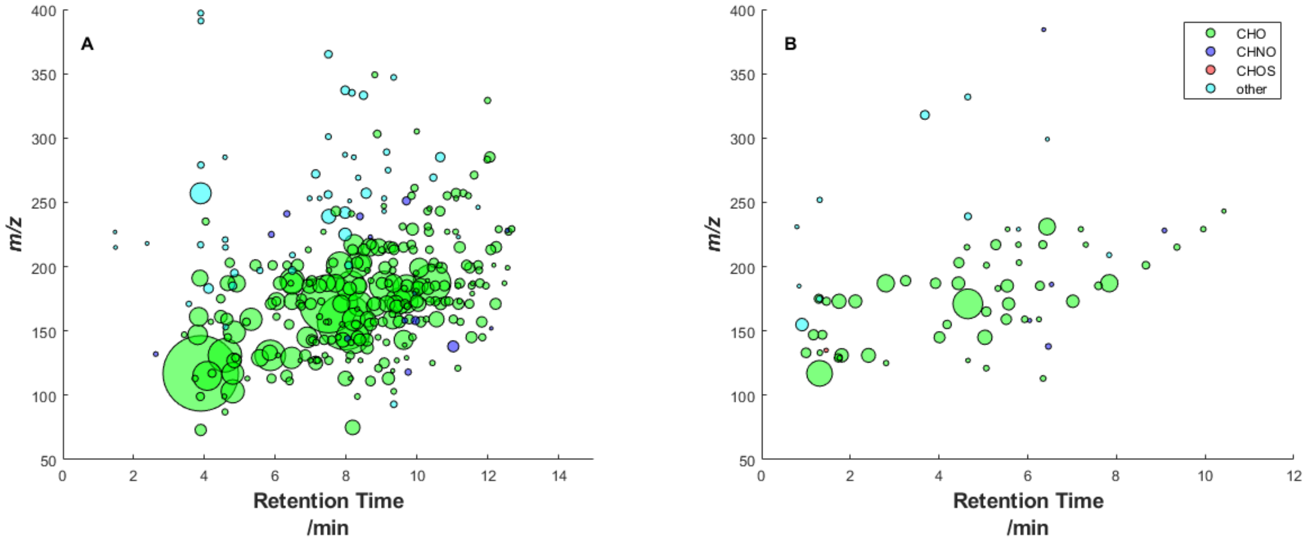

Figure S7 – Boxplot that shows the differences between unfrozen (n=4) and frozen (n=4) aliquots for the six targeted compounds for the 0.03 ng g^{-1} experiment (samples refrozen in glass vials). The dotted green line represents the procedural limit of detection.

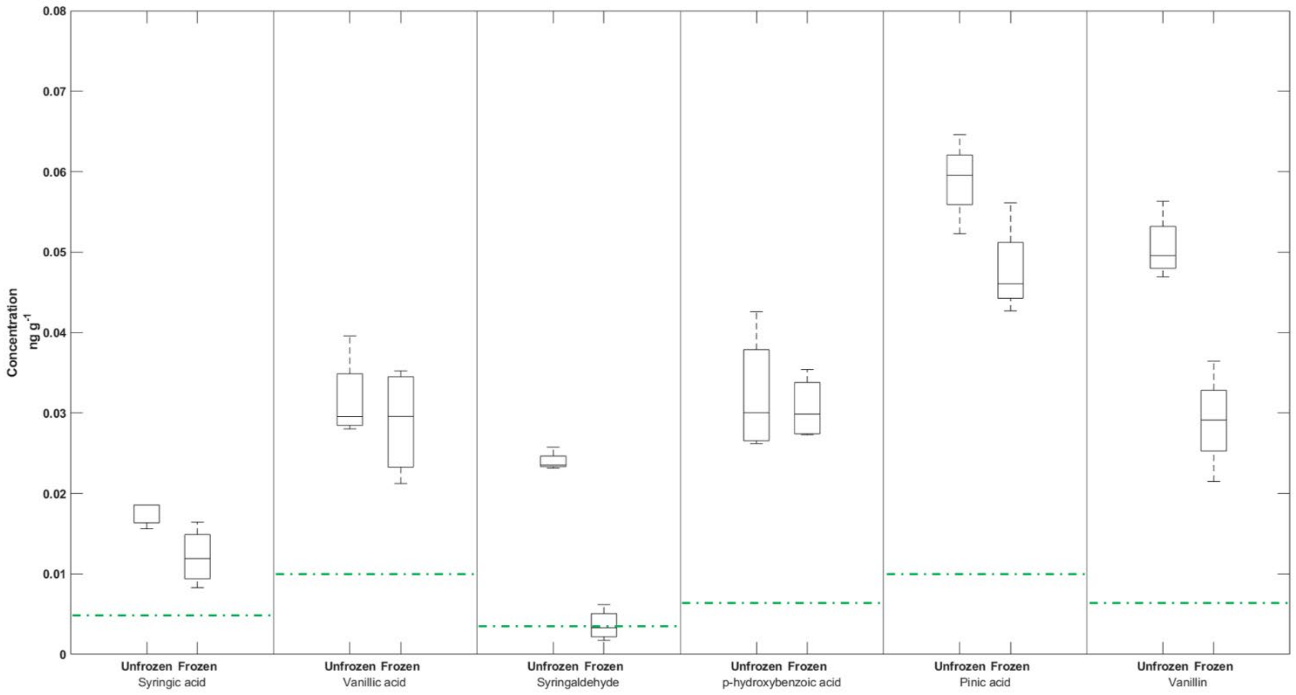

Figure S8 – Boxplot that shows the differences between Day 1 (n=4) and Day 2 (n=4) for the six targeted compounds for the 0.1 ng g^{-1} experiment (samples refrozen in glass vials). The dotted green line represents the procedural limit of detection.

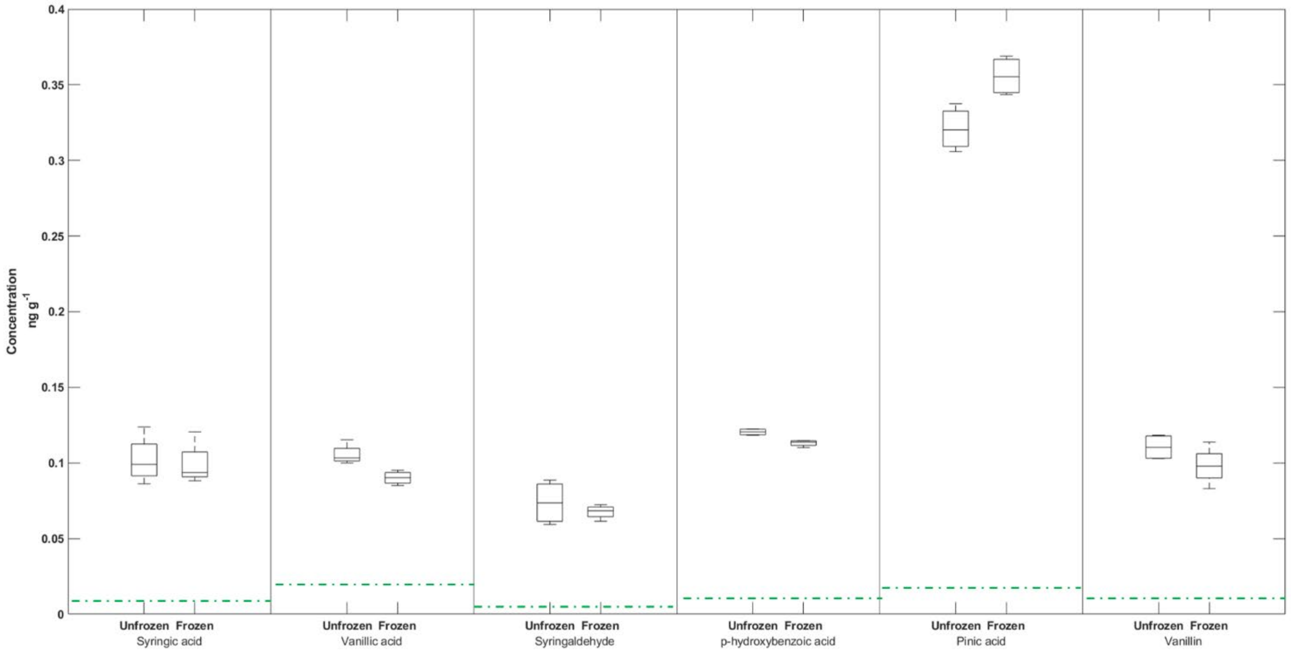

Figure S9 – Comparison between unfrozen (black solid lines) and frozen samples (red dashed lines) from the Colle Gnifetti ice core. Samples were frozen in the glass vials before extraction and they were all spiked to reach a final spiked concentration of ≈0.03 ng g^{-1}. Procedural limit of detection is given (green line).

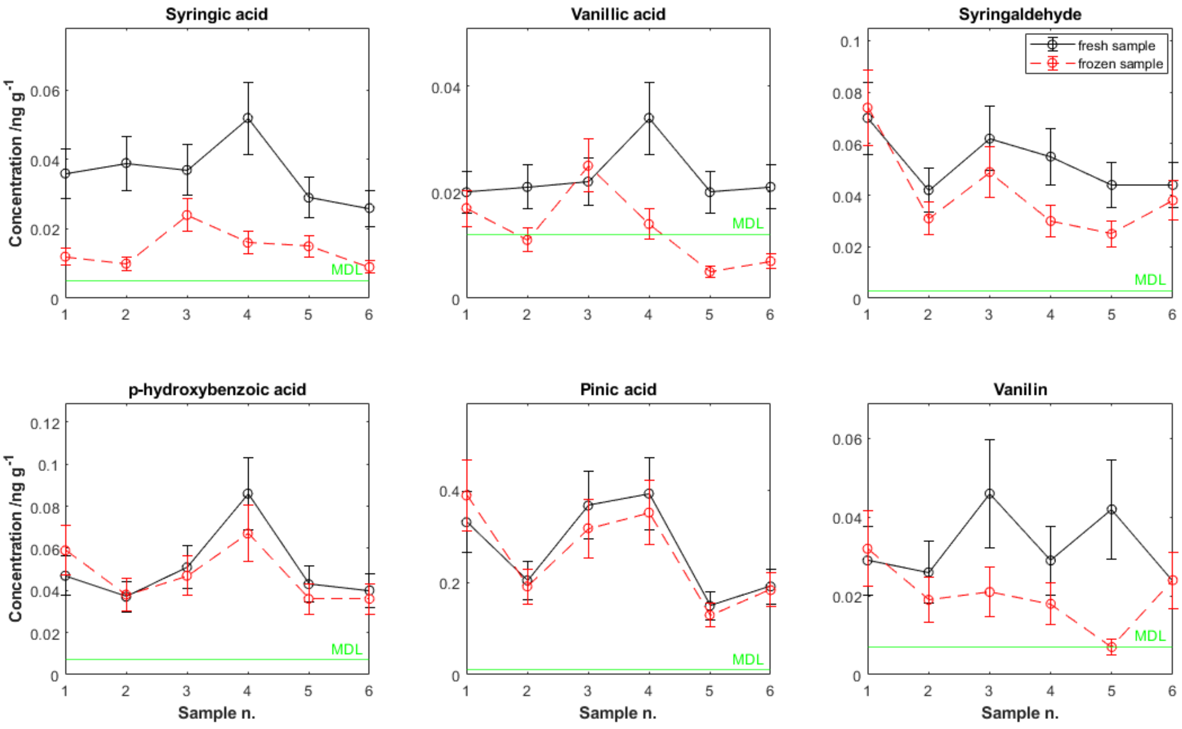

Figure S10 – Comparison between unfrozen (black solid lines) and frozen samples (red dashed lines) from the Colle Gnifetti ice core. Samples were frozen in the SPE cartridges before the elution and analysis, and they were all spiked to reach a final spiked concentration of ≈0.03 ng g^{-1}. Procedural limit of detection is given (green line). Sample 5 for syringic acid and vanillic acid is not reported due to an observed contamination.

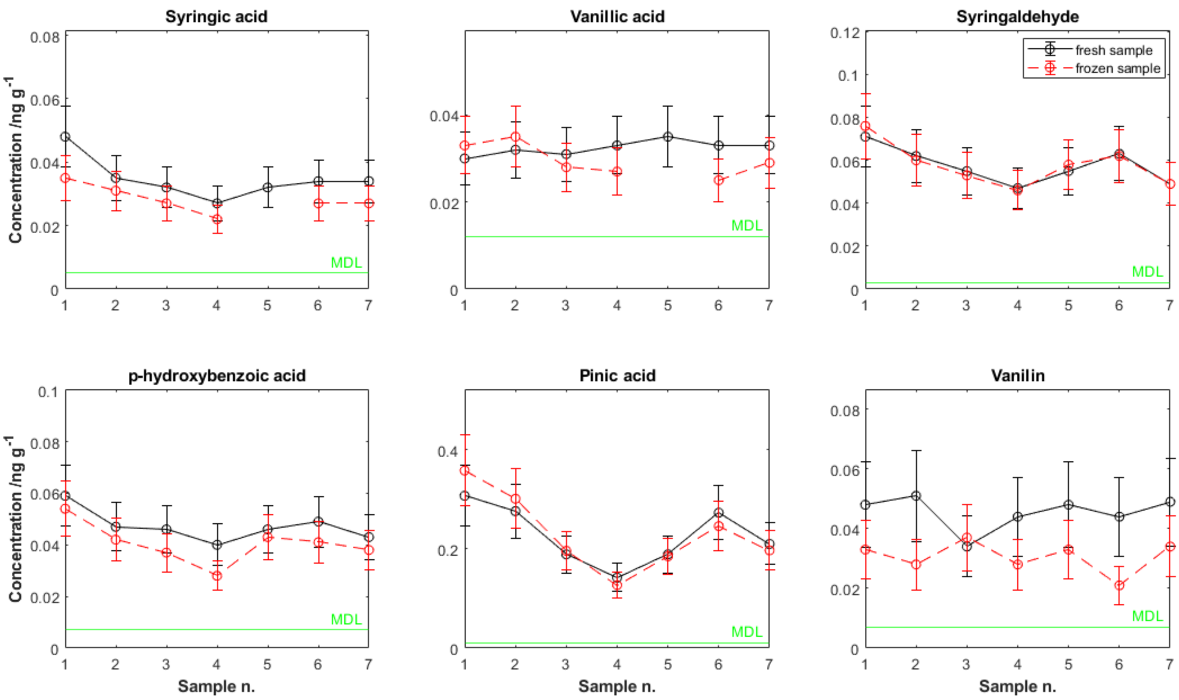

Figure S11 – The recovery of the six targeted species between frozen and non frozen cartridges is compared at three different concentrations (0.03 ng g^{-1}, 0.1 ng g^{-1} and 1 ng g^{-1}). Solid blue, yellow and red bars refer to recoveries from not frozen cartridges at 0.03 ng g^{-1}, 0.1 ng g^{-1} and 1 ng g^{-1}, respectively. Dashed blue, yellow and red bars refer to recoveries from frozen cartridges at 0.03 ng g^{-1}, 0.1 ng g^{-1} and 1 ng g^{-1}, respectively.

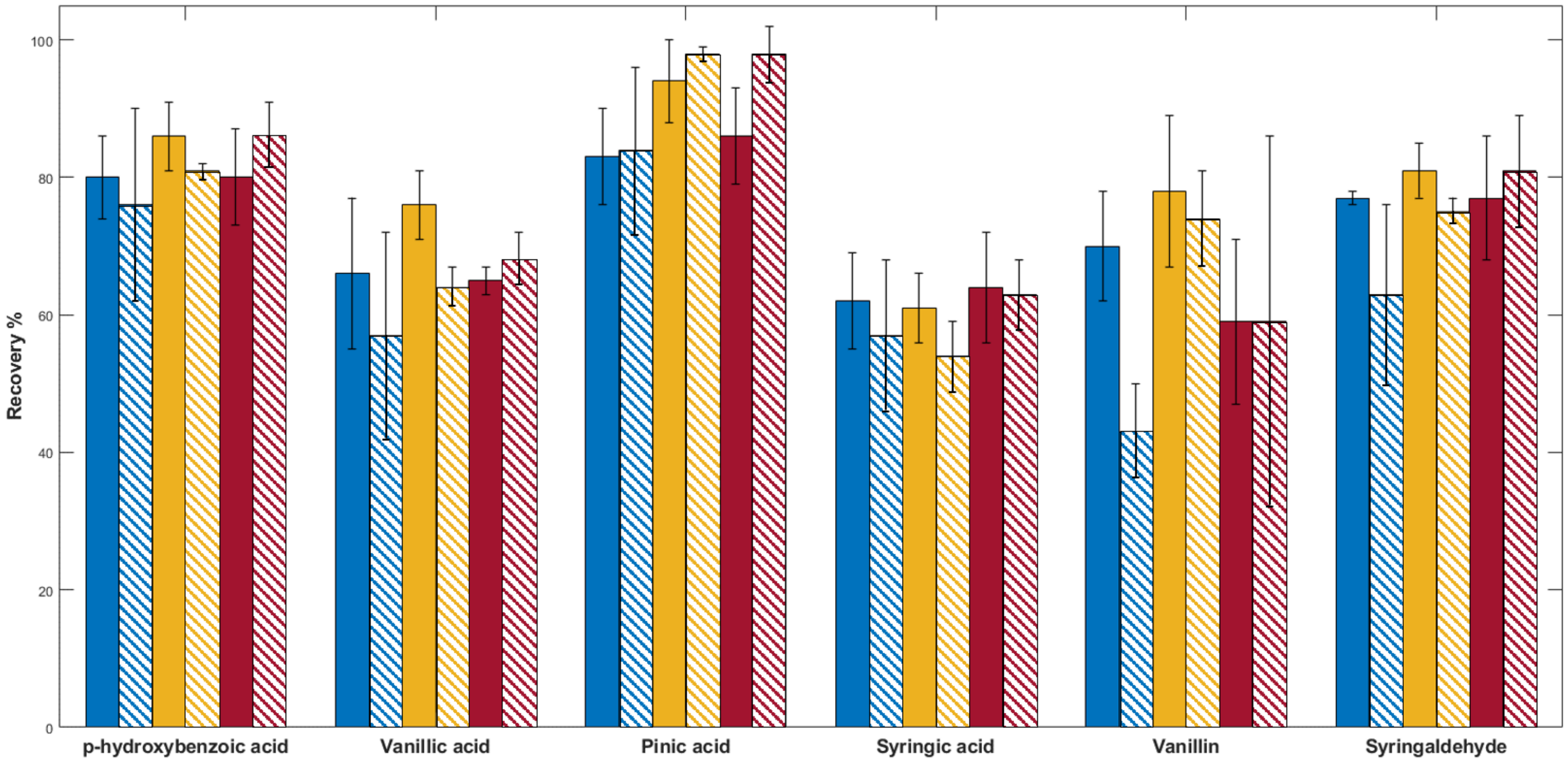

Figure S12 – Panel A): ratio between the area retrieved from samples frozen in the cartridges and the area of unfrozen samples. Panel B): ratio between the area calculated from samples frozen in the glass vials and the area of unfrozen samples. Only the compounds with ratios between 0.4 and 1.6 are reported. To see the entire distribution, refer to Figure S13.

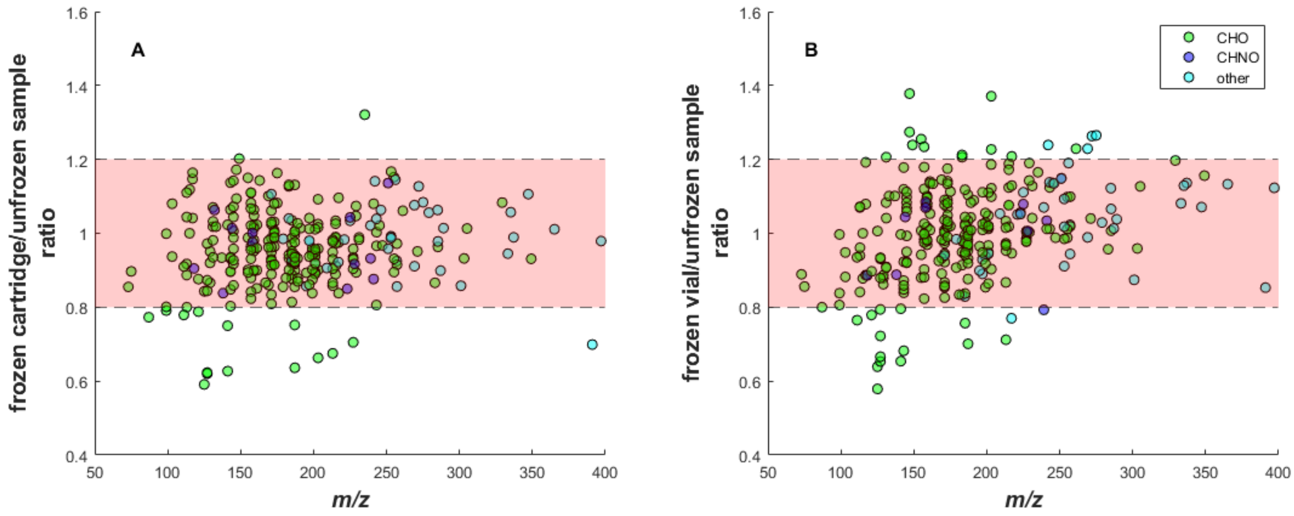

Figure S13 – Comparison between the number of compounds obtained in samples from frozen and unfrozen cartridges (panel A), and from frozen and unfrozen vials (panel b). More details are in the main text and in SI.4.

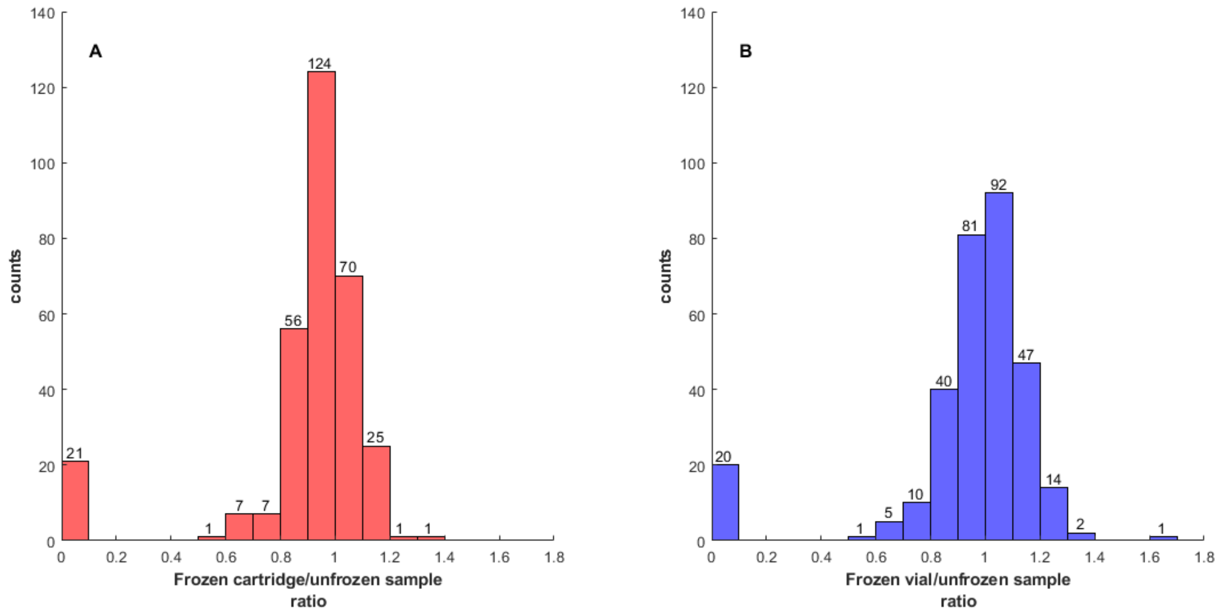

References

Barbaro, E., Feltracco, M., Spagnesi, A., Dallo, F., Gabrieli, J., De Blasi, F., Zannoni, D., Cairns, W. R., Gambaro, A., and Barbante, C.: Fast Liquid Chromatography Coupled with Tandem Mass Spectrometry for the Analysis of Vanillic and Syringic Acids in Ice Cores, Analytical Chemistry, 2022.

Gao, S., Liu, D., Kang, S., Kawamura, K., Wu, G., Zhang, G., and Cong, Z.: A new isolation method for biomass-burning tracers in snow: Measurements of p-hydroxybenzoic, vanillic, and dehydroabietic acids, Atmospheric Environment, 122, 142-147, 2015.

Grieman, M., Greaves, J., and Saltzman, E.: A method for analysis of vanillic acid in polar ice cores, Climate of the Past, 11, 227-232, 2015.

Grieman, M. M., Aydin, M., Isaksson, E., Schwikowski, M., and Saltzman, E. S.: Aromatic acids in an Arctic ice core from Svalbard: a proxy record of biomass burning, Climate of the Past, 14, 637-651, 2018.

Grieman, M. M., Aydin, M., Fritzsche, D., McConnell, J. R., Opel, T., Sigl, M., and Saltzman, E. S.: Aromatic acids in a Eurasian Arctic ice core: a 2600-year proxy record of biomass burning, Climate of the Past, 13, 395-410, 2017.

Kawamura, K., Izawa, Y., Mochida, M., and Shiraiwa, T.: Ice core records of biomass burning tracers (levoglucosan and dehydroabietic, vanillic and p-hydroxybenzoic acids) and total organic carbon for past 300 years in the Kamchatka Peninsula, Northeast Asia, Geochimica et Cosmochimica Acta, 99, 317-329, 2012.

McConnell, J. R., Edwards, R., Kok, G. L., Flanner, M. G., Zender, C. S., Saltzman, E. S., Banta, J. R., Pasteris, D. R., Carter, M. M., and Kahl, J. D.: 20th-century industrial black carbon emissions altered arctic climate forcing, Science, 317, 1381-1384, 2007.

Müller-Tautges, C., Eichler, A., Schwikowski, M., Pezzatti, G., Conedera, M., and Hoffmann, T.: Historic records of organic compounds from a high Alpine glacier: influences of biomass burning, anthropogenic emissions, and dust transport, Atmospheric chemistry and physics, 16, 1029-1043, 2016.

Steimer, S. S., Delvaux, A., Campbell, S. J., Gallimore, P. J., Grice, P., Howe, D. J., Pitton, D., Claeys, M., Hoffmann, T., and Kalberer, M.: Synthesis and characterisation of peroxypinic acids as proxies for highly oxygenated molecules (HOMs) in secondary organic aerosol, Atmospheric Chemistry and Physics, 18, 10973-10983, 2018.

Vogel, A. L., Lauer, A., Fang, L., Arturi, K., Bachmeier, F., Daellenbach, K. R., Käser, T., Vlachou, A., Pospisilova, V., and Baltensperger, U.: A comprehensive nontarget analysis for the molecular reconstruction of organic aerosol composition from glacier ice cores, Environmental Science & Technology, 53, 12565-12575, 2019.
